# A non-cell-autonomous actin redistribution enables isotropic retinal growth

**DOI:** 10.1371/journal.pbio.2006018

**Published:** 2018-08-10

**Authors:** Marija Matejčić, Guillaume Salbreux, Caren Norden

**Affiliations:** 1 Max Planck Institute of Molecular Cell Biology and Genetics, Dresden, Germany; 2 The Francis Crick Institute, London, United Kingdom; Institut Pasteur, France

## Abstract

Tissue shape is often established early in development and needs to be scaled isotropically during growth. However, the cellular contributors and ways by which cells interact tissue-wide to enable coordinated isotropic tissue scaling are not yet understood. Here, we follow cell and tissue shape changes in the zebrafish retinal neuroepithelium, which forms a cup with a smooth surface early in development and maintains this architecture as it grows. By combining 3D analysis and theory, we show how a global increase in cell height can maintain tissue shape during growth. Timely cell height increase occurs concurrently with a non-cell-autonomous actin redistribution. Blocking actin redistribution and cell height increase perturbs isotropic scaling and leads to disturbed, folded tissue shape. Taken together, our data show how global changes in cell shape enable isotropic growth of the developing retinal neuroepithelium, a concept that could also apply to other systems.

## Author summary

As the shape of a tissue or organ is usually important for its function, it is critical to understand both how tissues form with the correct size and how their shape is maintained during development. However, so far, most studies have focused on how the shape of tissues changes during morphogenesis, while little is known about how tissues keep their shape during growth. Many tissues, including the human nose and retina, establish their shape early in development and need to maintain this shape as the tissue grows. To understand how shape is maintained during growth, cellular behaviors underlying isotropic growth need to be linked to overall tissue parameters. To achieve this, 3D studies of entire tissues, with cellular resolution, are needed. This study took advantage of the exceptional imaging possibilities of the developing zebrafish retina to accomplish this goal. It identified cell elongation as the key cellular shape change that allows the retinal tissue to maintain its shape during growth. This 3D analysis during development, together with a theoretical description, shows that spindle-like retinal cells elongate further thanks to a timely non-cell-autonomous redistribution of the cytoskeletal element actin. Overall, this study provides a fascinatingly simple example of how isotropic growth of a tissue is achieved and, at the same time, showcases the severe consequences on tissue geometry that arise if cellular shape changes do not occur at the correct developmental time.

## Introduction

**Fig 1:**
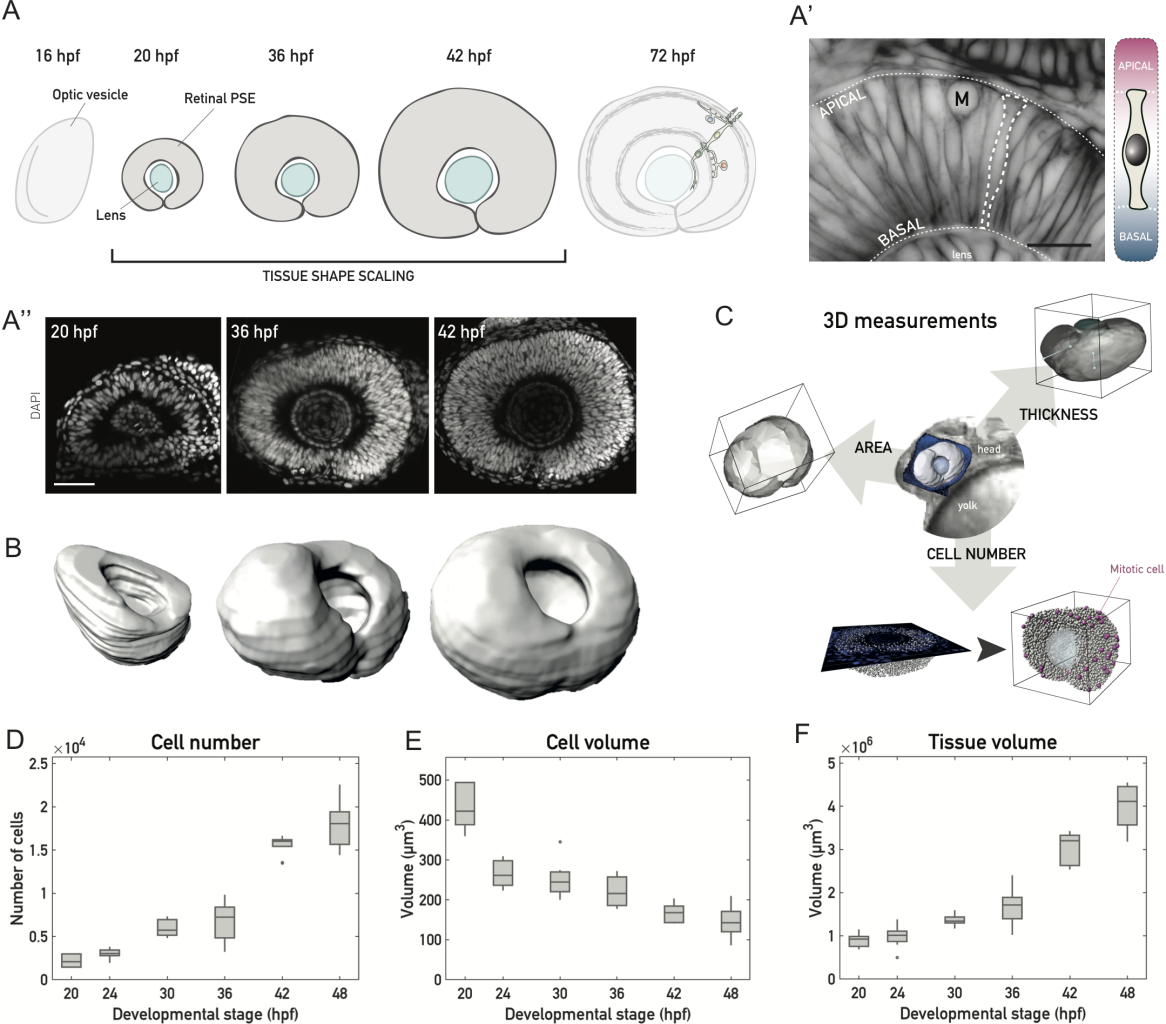
A 3D tissue-wide analysis allows cell-level investigation of tissue shape maintenance during vertebrate retinal PSE growth. (A) Schematic of vertebrate retinal development. After the optic vesicle forms the optic cup, cells in the retinal PSE proliferate as the tissue maintains its shape (20–42 hpf) to ultimately give rise to the laminated neuronal retina. (A’) The developing vertebrate retina is a PSE. Left: Optical slice through the retinal PSE at approximately 30 hpf, with a single cell outlined (dashed white line). Apical and basal surfaces of the tissue are outlined (dashed white lines). Cell membranes are labeled by Tg(actb1::HRAS-EGFP). Scale bar: 20 μm. Right: Schematic of a cell in the retinal PSE. The apical endfoot is shown at the top, the basal endfoot at the bottom (thin white dashed lines). (A”) Representative images of the retinal PSE in stages of proliferative growth. Nuclei are labeled with DAPI. Scale bar: 50 μm. (B) Examples of 3D surfaces for the retinal PSE tissue-wide growth analysis, shown for 20 hpf, 36 hpf, and 42 hpf. Surfaces were manually segmented, rendered, and analyzed using Imaris 8.3 (Bitplane). Related to S1 Movie. (C) Three exemplary parameters (tissue surface area, thickness, cell number) analyzed in the 3D characterization of size and shape of the retinal PSE (see Materials and methods). Middle: brightfield image of the anterior zebrafish body, with the head and a portion of the yolk sac visible. A 3D surface of the retinal PSE, segmented out from the surrounding tissue (blue), is overlaid to the brightfield image for orientation. In the representation of the tissue thickness (cell height) analysis, the light blue lines mark 2 (out of 5) positions at which thickness was measured in 3D. In the representation of the cell number analysis, mitotic cells (“M”) are visualized as magenta spheres and all other cells (interphase) as white spheres. (D) Total cell number increase during retinal PSE growth. (E) Cell volume decrease, as measured and corrected from mitotic cell volumes. (F) Tissue volume increase from manually segmented retinal tissues. *N* = 10 samples/stage for all plots. On boxplots, the central mark marks the median, the bottom and top box limits indicate the 25th and 75th percentile, respectively, the whiskers indicate the most extreme data points that are not considered outliers, and outliers are plotted as points. (Underlying data can be found at DOI: 10.5281/zenodo.1316912; /Matejcic-et-al_2018/Data/F1_2_3D_S12BD34.csv.). hpf, hours post fertilization; PSE, pseudostratified epithelium.

Acquiring the correct size and shape during development is a crucial prerequisite for tissue and organ function. In diverse tissues, such as the *Drosophila* salivary gland [1] or the vertebrate retina [2], shape characteristics are established early in development and need to be retained throughout growth. This necessitates an isotropic rescaling of the initial tissue shape (Fig 1A). How such uniform, isotropic rescaling is achieved through cell and tissue level processes, however, is not yet well explored.

Nevertheless, some of the molecular factors governing cell and tissue geometry have been extensively studied. Both microtubule reorganization and cortical actomyosin rearrangements can influence shapes of individual epithelial cells, as well as the shape of whole tissues [3–10]. When studying these factors, most investigations, so far, explored anisotropic tissue shape changes occurring during development, while isotropic growth and shape maintenance are not as well understood. In addition, previously, tissue growth and shape were primarily explored in two dimensions [5,9,11–13]. Thus, a thorough evaluation of how tissue growth is coordinated by cell and tissue parameters in 3D is currently lacking.

One tissue that allows analyzing cell and tissue shape during growth is the curved vertebrate retinal neuroepithelium. The retinal neuroepithelium establishes its shape early in development by formation of the optic cup [2,14] and grows continuously afterwards (Fig 1A). During this proliferative growth, the neuroepithelium is pseudostratified [15], with cells attached to both the apical side and the basal lamina. Nuclei are located along the apicobasal axis, except in mitosis, as all cells divide apically [16]. Such pseudostratified epithelia (PSE) are evolutionarily conserved and serve as organ precursors in a wide range of species [15]. In the retinal neuroepithelium, as well as in most other PSE, cells progressively increase their height as the tissue proliferates and grows [15,17–19]. So far, however, it is not understood what drives this cell elongation over development and whether and how it is linked to the overall tissue growth and shape maintenance.

To investigate how growth and shape coordinate in the developing retinal neuroepithelium, we used zebrafish to analyze cell- and tissue-wide shape changes during proliferative growth. We generated a quantitative tissue-wide 3D dataset, which allowed us to break down 3D growth into individual cellular components through balance equations. Our analysis revealed that isotropic growth relies on apicobasal cell elongation. This cell elongation is governed by cellular actin redistribution that occurs in a non-cell-autonomous manner. Furthermore, cell elongation occurs in concert with continued proliferation and needs an unperturbed extracellular matrix (ECM). Our data are validated by a simplified theoretical model and balance equations that account for the main aspects of tissue changes. If actin redistribution and thereby cell height increase is impaired, tissue shape maintenance does not take place, and the tissue gets distorted upon further growth.

## Results

### During retinal PSE growth, single-cell aspect ratios change, while the overall tissue aspect ratio is maintained

In order to understand how cell and tissue shape changes are linked during growth of the proliferating retinal PSE (Fig 1A and 1B), we quantified cell and tissue geometry of the retinal neuroepithelium over development.

By performing tissue-wide 3D analysis (Fig 1B and 1C and S1 Movie), we analyzed growth in 4–6 h intervals between 20 h post fertilization (hpf), when morphogenesis of the optic cup is complete, and 48 hpf, a stage when neuronal differentiation is ongoing, (Fig 1A and 1B, 10 embryos/time point). For details of the analysis, we refer the reader to the Materials and methods part. In brief, the whole volume of the tissue and its apical surface were manually outlined in 3 dimensions. These outlines were used to obtain 3D tissue segmentations, using Imaris, and subsequently allowed for analysis of cell number, size, and shape in the entire retinal PSE (Fig 1C).

While apoptosis was negligible between 20 hpf and 48 hpf (S1 Fig) [20], the total cell number increased 8-fold, from about 2,200 to about 18,000 (Fig 1D, S1 Fig). Concurrently, single cell volumes decreased by almost two-thirds, from 440 to 140 μm^3^ (Fig 1E). Overall, the tissue volume increased exponentially by a factor of approximately 4.4 over 28 h, with a rate of about 0.055/h and a volume doubling time of 12.5 h (Fig 1F).

**Fig 2:**
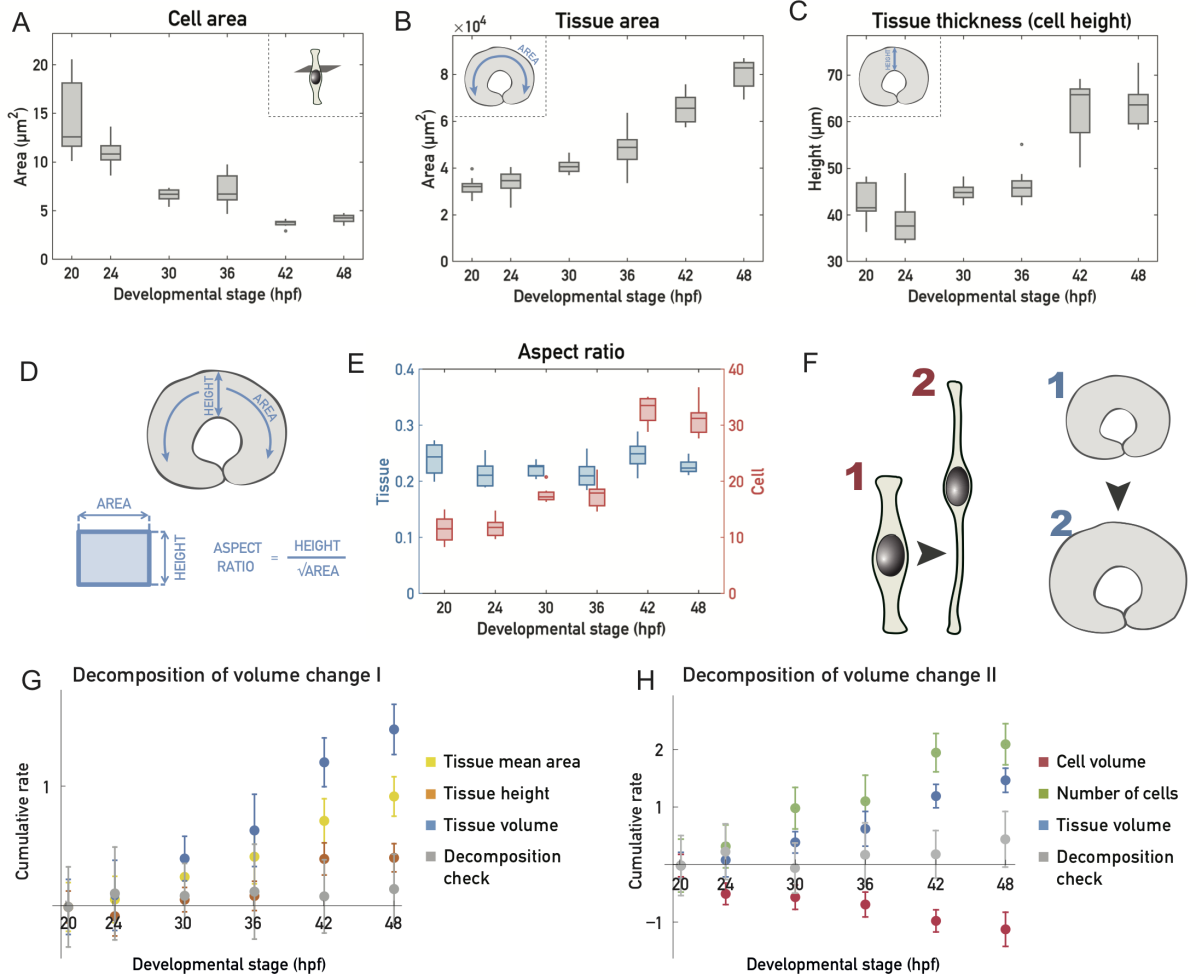
Cell shapes change while tissue shape is maintained during growth of the retinal PSE. (A) Mean cell area decrease. Cross-sectional cell area is calculated as an average of cell apical and basal cell endfeet areas, calculated from cell number and tissue areas. (B) Mean tissue surface area increase. Surface area is calculated as an average of the apical and basal tissue surface areas measured tissue-wide in 3D. (C) Mean tissue thickness increase as measured by cell height. Thickness is measured in 3D in 5 representative regions of the tissue (see Materials and methods). (D) Aspect ratios were calculated by dividing the tissue height (plotted in C) by the square root of the average tissue area (plotted in B) or average cell area (A). (E) Tissue (blue) and cell (red) aspect ratios. (F) Schematic of the tissue shape during retinal PSE growth. PSE cells change their shape by elongating and becoming thinner, while the overall tissue shape remains unchanged. (G) Cumulative logarithmic rate of change for the mean tissue area (yellow), tissue height (orange), and tissue volume (blue). Gray data points correspond to the sum of these 3 independently measured rates of changes, which add up to 0 within experimental standard deviation. (H) Cumulative logarithmic rate of change for the cell volume (red), number of cells (green), and tissue volume (blue). Gray data points correspond to the sum of these 3 independently measured rates of changes, which add up to 0 within experimental standard deviation. *N* = 10 samples/stage for all plots. (Underlying data can be found at DOI: 10.5281/zenodo.1316912; /Matejcic-et-al_2018/Data/F1_2_3D_S12BD34 .csv.). hpf, hours post fertilization; PSE, pseudostratified epithelium.

In addition to decreasing their volume, cells changed their shape, becoming thinner (Fig 2A) and more apicobasally elongated (Fig 2C).

The largest change in aspect ratio and thereby cell shape occurred between 36 hpf and 42 hpf (Fig 2D and 2E), as did the largest increase in cell numbers (Fig 1D), suggesting differences between an early (before 36 hpf) and later growth phase (after 36 hpf). We next examined the overall changes in tissue area (Fig 2B) and height (Fig 2C) over time. Before 36 hpf, tissue height stayed fairly constant (Fig 2C), and most of the volume growth was accounted for by area expansion (Fig 2B and 2G). After 36 hpf, however, a significant increase of tissue height was seen, which accounted for a larger fraction of tissue growth (Fig 2G). While single cells elongated and changed their aspect ratio (Fig 2E and 2F), the tissue area (Fig 2B) and thickness (Fig 2C) roughly scaled with each other, such that the aspect ratio of the tissue remained constant (Fig 2D and 2E). This may seem in contradiction with the observation that area expansion occurs at constant height before 36 hpf. However, during this phase, the area expansion is small enough (Fig 2B) for the tissue aspect ratio not to be significantly affected (Fig 2E).

Overall, our measurements indicate that, while cell shape changes, height and area expansion of the retinal PSE occur concurrently to maintain a fixed tissue aspect ratio.

### Retinal PSE growth is not strongly oriented and homogeneous

**Fig 3:**
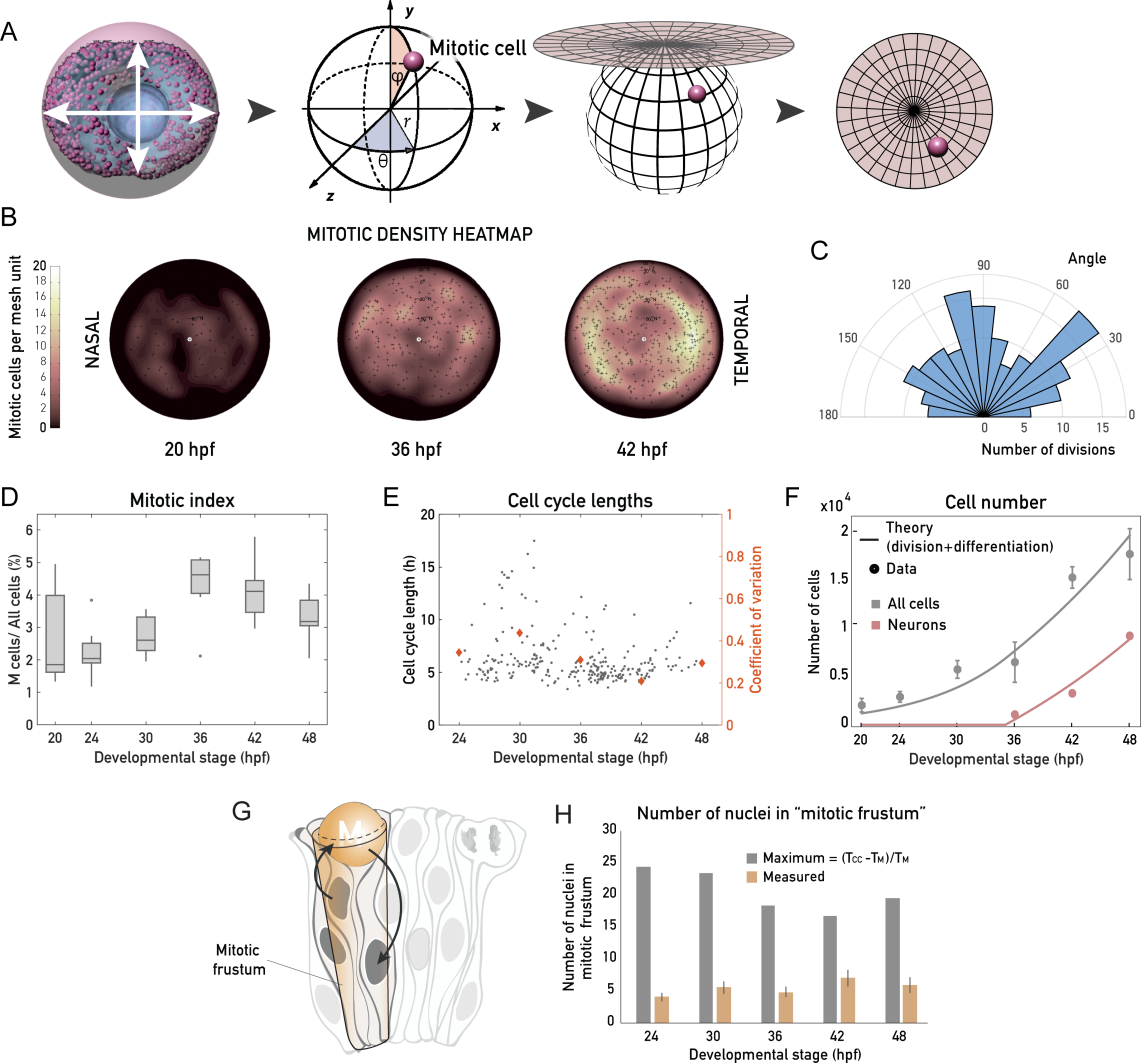
Retinal PSE growth is not strongly oriented, is homogeneous, and is unconstrained by the apical surface area. (A) Schematic of the mitotic distribution workflow to generate 2D mitotic density heatmaps; 3D Cartesian coordinates of every mitotic cell (see Materials and methods) were transformed into polar, spherical coordinates, which were then projected into 2 dimensions using a density-preserving azimuthal projection tool. (B) Typical 2D heatmaps of mitotic densities at 20 hpf, 36 hpf, and 42 hpf, obtained by transformations in (A). *N* = 10 samples/stage. (C) Rose plot of division angles, analyzed from samples 24–42 hpf. *N* = 15 embryos. Related to S2 Movie. (D) The mitotic index through development, calculated as the fraction of mitotic cells of all retinal PSE cells in a 3D tissue-wide retinal sample. (E) Cell cycle lengths of progenitor retinal PSE cells, analyzed by manual tracking of 254 cells from 20 embryos. Related to S3 Movie. The developmental stage on the x-axis is the middle point of the cell cycle, in hpf. Times on the x-axis are the CoVs for each stage plotted as orange diamonds (right y-axis). Data for CoV were binned as stage ± 3 h. (F) Total number of cells in the retina (gray points, as in Fig 1D) and number of committed progenitors/neurons (pink points) from data in S2 Fig. Gray and pink lines: theoretical cell and theoretical neuron number, assuming a constant rate of division and a 35% probability of dividing progenitors to produce 2 committed progenitors/neurons after 35 hpf (see S1 Text). Data points are plotted as mean ± SD. (G) Schematic representation of PSE tissue architecture, with apical mitoses (see also S3 Fig), migrating nuclei (arrows), and the mitotic frustum. The mitotic frustum is depicted as a truncated conical unit below the rounded mitotic cell. (H) Number of cells under the rounded mitotic cell. Measured values are calculated from tissue-wide nuclear density and mitotic frustum volume in each developmental stage (*N* = 10 samples/stage, see Materials and methods). Maximal possible number of cells is calculated as the time during which nuclei are absent from the apical surface (T_CC_ − T_M_), divided by T_M_ in each stage (see Materials and methods). (Underlying data can be found at DOI: 10.5281/zenodo.1316912; for panel B at /Matejcic-et-al_2018/Tools/MitoticDistribution/Positions_data/, for panel D at /Matejcic-et-al_2018/Data/F1_2_3D_S12BD34.csv, for panel E at /Matejcic-et-al_2018/Data/F3E.csv, panel H at /Matejcic-et-al_2018/Data/F3H.csv and F3H_4A.csv. The theoretical analysis for panel F can be found at /Matejcic-et-al_2018/Theory/Data analysis_essentials.nb.). CoV, coefficient of variation; hpf, hours post fertilization; PSE, pseudostratified epithelium; T_CC_, total cell cycle time; T_M_, duration of mitosis.

The fact that the retinal PSE appears to grow homogeneously suggested that mitotic events are distributed homogenously and without a preferred direction in the apical plane of the epithelium. To test this notion, we analyzed the distribution of mitosis throughout the proliferative phase using a custom tool (Fig 3A and Materials and methods) that allowed the projection of the 3D coordinates of all apically dividing cells onto a 2D density heatmap (Fig 3B).

This analysis confirmed that mitotic cells were distributed evenly across the apical surface at all developmental stages (Fig 3B), making proliferative growth homogeneous. Cell division angles in the plane of the epithelium were observed by live imaging and did not show a strong alignment along a preferred direction (Fig 3C and S2 Movie).

Our mitotic density heatmaps (Fig 3B) in combination with the mitotic index analysis (Fig 3D) showed that mitotic activity peaked at approximately 36–42 hpf. To understand whether this mitotic peak was due to an increased mitotic duration or due to overall cell cycle shortening, we analyzed cell cycle length by live imaging of a mosaically expressed nuclear marker, using light sheet microscopy to reduce phototoxicity (S3 Movie) [21]. Manual cell tracking (*N* = 250, 20 embryos) revealed that, as the length of mitosis remained constant (S2 Fig), average cell cycle lengths shortened from 7.3 h at 30 hpf to 5.3 h at 42 hpf (Fig 3E) and became less variable, as seen by the reduced coefficient of variation (Fig 3E). Thus, the observed peak in proliferation between 36 hpf and 42 hpf resulted from an overall cell cycle shortening.

The cell cycle length analysis in the proliferative growth phase revealed that the rate of cell proliferation varied around an average value of approximately 0.11 cell divisions per cell per hour (Fig 3E, S4 Fig). Furthermore, our measurements showed that at 48 hpf, about 18,000 cells inhabit the retina (Fig 1D). However, if all cells were continuously dividing at an average rate of 0.11/h starting from 2,177 cells at 20 hpf, the number of cells at 48 hpf should reach about 47,000. To understand this discrepancy in cell number at 48 hpf, we analyzed the fraction of cells that exited the cell cycle. We assessed the number of differentiating cells between 36 hpf (the stage when neurogenesis starts [22]) and 48 hpf (S2 Fig) using fluorescence-activated cell sorting (FACS) of the SoFa transgenic line, which labels all emerging retinal neurons [23]. A model in which progenitors divide at a constant rate of 0.11/h then enabled us to account for the number of neurons and total number of cells. In this model, all divisions give rise to progenitors prior to 35 hpf, while 35% of the divisions give rise to committed precursors/neurons [22,24] after 35 hpf (Fig 3F and S1 Text). Using both cell division and differentiation rates calculated from our data, one can account for the total number of cells at different developmental stages of the retinal PSE (Fig 3F).

We next used tissue volume, cell volume, and cell number measurements to decompose the volume change in the tissue into (i) changes of average cell volume and (ii) changes in the number of cells through cell division. The cumulative logarithmic rates of change of these quantities are shown in Fig 2H. We verified that the sum of different contributions adds up to 0 within the experimental standard deviation. We found that, because the rate of volume growth is about twice as low as the relative rate of cell number increase, the average cell volume is decreasing at roughly the same relative rate as the tissue volume is increasing (Fig 2H).

Taken together, our data show that overall, the retinal PSE increases its volume homogeneously and without a strong preferred direction in the plane of the epithelium, and the cell division rate is about twice as large as the rate of volume growth, leading to a decrease in average cell volume before 35 hpf.

### Retinal PSE growth is not limited by apical surface availability

The combined decrease in cell volume and increase in cell number lead to increased cell density in the retinal PSE (Fig 1A”, S2 Fig). We wondered whether this increase in cell density could influence proliferative growth of the retina. Such a hypothesis was posed in the 1970s, in which it was postulated that proliferative PSE growth can be constrained by a self-inflicted proliferative trap [17]. It was suggested that this trap arises from the fact that all cell divisions occur exclusively at the apical surface, where all PSE cell nuclei migrate prior to division [15,17,25]. When too many nuclei are packed under the apical surface, or too many cells divide simultaneously, the availability of the apical surface was hypothesized to become a spatial limitation to further proliferation (Fig 3G, S3 Fig). To this date, however, we are not aware of an experimental dataset that allowed to test this idea. With our quantitative dataset at hand (Figs 1 and 2), we first analyzed the fraction of the apical surface occupied by rounded mitotic cells between 20 hpf and 48 hpf. This analysis showed that mitotic cells never occupied more than 20% of the total apical surface, even at times of highest mitotic activity (S3 Fig). We then calculated a theoretical maximal number of proliferative cells in the conical volume beneath a single mitotic cell (Fig 3H), given its cell cycle parameters, and compared this value to the number of cells stacked beneath a dividing cell at the apical surface in our measurements. Again, the measured numbers were consistently far from the calculated theoretical maximum throughout development. Together, these results show that no proliferative trap exists in the growing retinal PSE and that proliferative retinal growth is not constrained by the availability of the apical surface. With this, we provide, to the best of our knowledge, the first measurements of PSE nuclear packing in relation to its proliferation through development and show that the suggested proliferate trap [17,19,25,26] is not a general phenomenon that applies to all pseudostratified tissues.

### A basolateral actin bias establishes a nuclear exclusion zone

**Fig 4:**
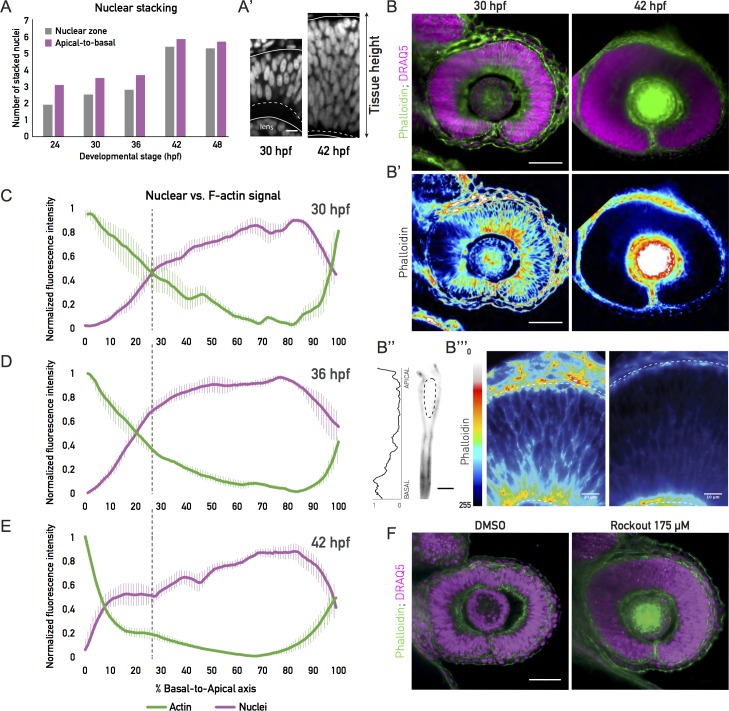
A tissue-wide basolateral actin accumulation establishes a basal nuclear exclusion zone. (A) Number of stacked nuclei along the apicobasal axis of the retinal PSE. Number in the nuclear zone was calculated from nuclear stainings by dividing the thickness of the zone of the nuclear signal by the average long axis of the nucleus. Apical-to-basal number of layers was calculated as total tissue height divided by the average nuclear long axis. (A’) Image of the basal nuclear exclusion zone in the first growth phase (30 hpf; square bracket, dashed line) and its absence in the second growth phase (42 hpf; dashed line). Solid lines mark the apical and basal tissue surface. Nuclear staining: DRAQ5. Scale bar: 10 μm. (B) Tissue-wide basolateral actin accumulation (green) and the nuclear zone (magenta) at 30 and 42 hpf. (B’) Lookup table indicates minimal and maximal phalloidin signal values from (B). Scale bar in (B) and (B’): 50 μm. (B”) Phalloidin signal in a manually segmented PSE cell, showing basolateral accumulation on the single-cell level. Dashed line outlines the cell nucleus. Scale bar: 5 μm. Left: Normalized basal-to-apical phalloidin signal intensity along the lateral cell membrane of cell on right. (B”’) Zoom-in on the apicobasal tissue axis in (B’). Dashed lines indicate apical and basal tissue surfaces. Lookup table indicates minimal and maximal phalloidin signal values. Scale bar: 10 μm. (C), (D), (E) Normalized average intensity distributions of phalloidin (green) and DRAQ5 (magenta) signal in the tissue volume along the apicobasal axis of the retinal PSE at 30 hpf (C), 36 hpf (D), and 42 hpf (E). Dashed black lines indicate border of basal exclusion zone and actin accumulation at 30 hpf. Values in each sample are normalized to minimum and maximum values. Data are shown as mean ± SEM; 5–9 samples/stage. (F) Rockout-treated and 0.3% DMSO-treated retinal PSE at 36 hpf. Rockout treatment (175 μM) was started at 30 hpf (see S6 Movie). (Underlying data can be found at DOI: 10.5281/zenodo.1316912; for panel A at /Matejcic-et-al_2018/Data/F3H_4A.csv, for panels C-E at /Matejcic-et-al_2018/Data/F4CDE_6BC.csv.). DRAQ5, deep red anthraquinone 5; hpf, hours post fertilization; PSE, pseudostratified epithelium.

While proliferative growth was not constrained by the apical surface, the number of nuclei stacked in the tissue nevertheless increased during the proliferative phase (Fig 4A and 4A’).

We analyzed this increase in nuclear stacking throughout development by dividing the length of the apicobasal axis containing nuclear signal by the average nuclear length (Fig 4A and 4A’). Between 36 hpf and 42 hpf, nuclear stacking increase was most prominent, with the average number of stacked nuclei rising from 2.8 to 5.5 nuclei, respectively (Fig 4A, gray bars).

The length of the apicobasal nuclear zone (Fig 4A), however, differed from the tissue thickness assessed in our initial growth analysis (Fig 2C). One explanation for this discrepancy could be that nuclei do not occupy the entire length of the tissue. Inspecting the tissue in more detail, we indeed noted that, prior to the significant increase in nuclear stacking, the basal zone of the retinal PSE was devoid of nuclei (Fig 4A’). This resulted in a shorter nuclear zone compared to the entire tissue thickness (Fig 4A’). This nuclear exclusion zone was diminished by 42 hpf, the time at which the major increase in nuclear stacking is observed. From this stage on, nuclei occupied the entire apicobasal tissue axis (Fig 4A and 4A’).

We next asked how nuclei might be restrained from occupying the basal region before 42 hpf. It was previously reported that a basolateral actin accumulation is present in cells of the early zebrafish optic cup [2]. We therefore asked whether this actin accumulation persisted at later stages and whether it might be linked to the basal nuclear exclusion zone (Fig 4B). To this end, we analyzed the distribution of actin signal intensity along the apicobasal axis of the cells, in relation to nuclear distribution (Fig 4C–4E). Before 40 hpf, the basolateral actin accumulation was consistently observed (Fig 4B–4D). More specifically, at 30 hpf, the basolateral actin accumulation took up 26% of the total cell height, or an average 12 μm of the apicobasal axis (Fig 4C), both on the single cell (Fig 4B”) and tissue-wide level (Fig 4B, 4B’ and 4B”’). However, at 36 hpf, the accumulation started to disappear (Fig 4D), spanning an average 20% of the cell axis (9 μm). At 42 hpf, the basolateral actin pool was drastically reduced (Fig 4B, 4B”’ and 4E), occupying only the most basal 8% (5 μm) of the cells’ apicobasal axis. At all stages, this tissue-wide basolateral actin accumulation was in precise negative correlation with the nuclear exclusion zone (Fig 4C–4E).

To test whether this actin accumulation was indeed responsible for the exclusion of nuclei from basal positions, we abolished the basolateral actin at 30 hpf using the Rho-kinase (ROCK) inhibitor, Rockout [2,27]. This treatment led to a disappearance of both the actin accumulation (Fig 4F) and the basal nuclear exclusion zone, as nuclei were consequently occupying the entire apicobasal axis (Fig 4F).

Thus, a basolateral actin accumulation creates a basal nuclear exclusion zone, and only once it disappears nuclei occupy basal positions in the tissue.

### Actin redistribution is linked to an increase of cell height

**Fig 5:**
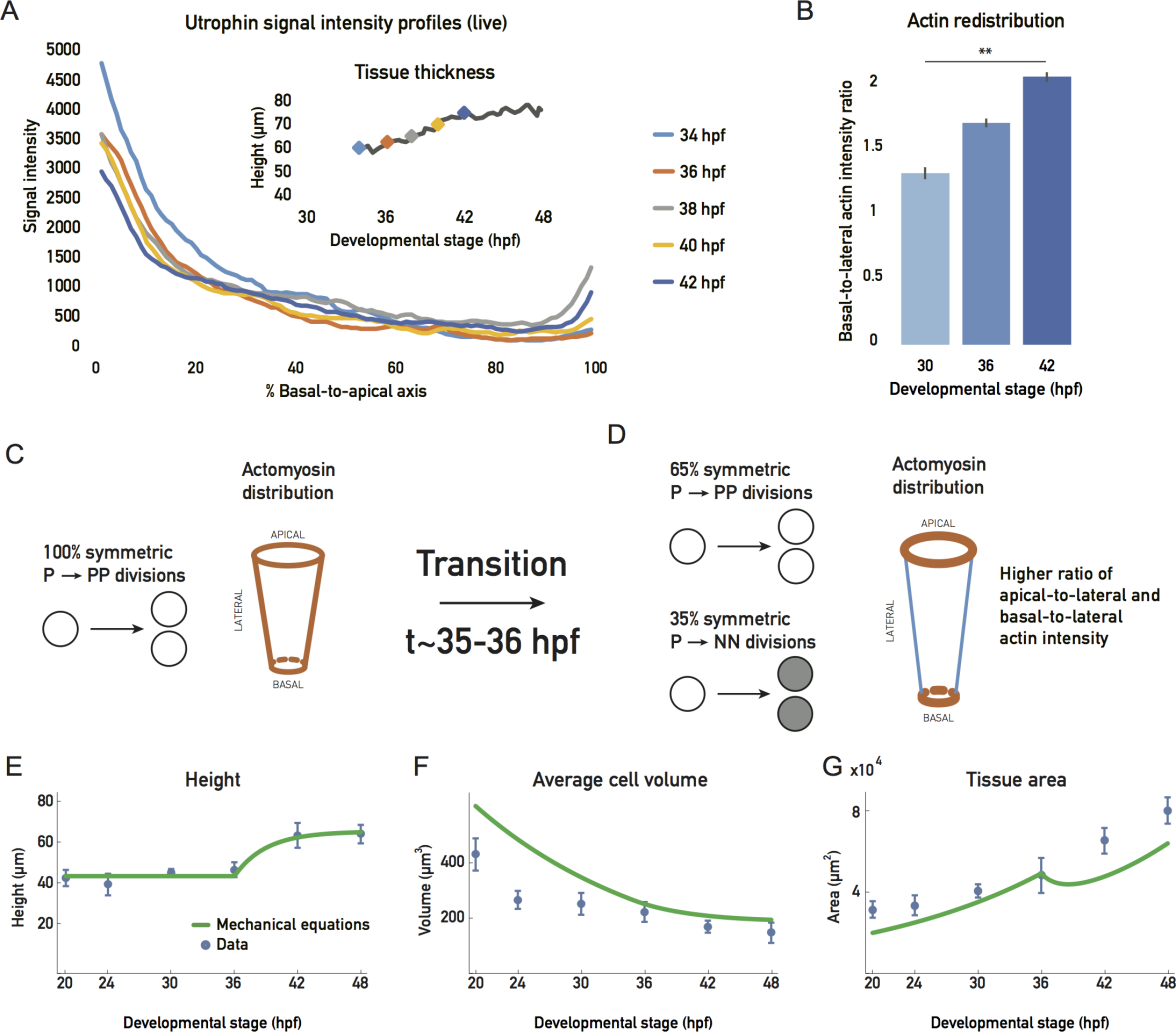
Basolateral actin redistribution coincides with cell height increase. (A) Apicobasal actin intensity distributions from live Tg(actb1::GFP-UtrophinCH) embryo measurements over development. Inset plot shows cell height increase in the same sample, with actin intensity measurement points marked (color code matches main plot) (S7 Movie). (B) Actin redistribution over development. Ratios of basal-to-lateral phalloidin signal; 5 samples/stage. Mean ± SD. Mann-Whitney test, *p*-value 0.0079. (C), (D) Schematic of the simplified theoretical description of retinal growth. Prior to about 35–36 hpf, all progenitor divisions give rise to 2 progenitors, and a constant ratio of apicobasal line tension to lateral surface tensions is maintained. (D) After this transition, 35% of the divisions give rise to committed progenitors or neurons, and a redistribution of actin drives a change in the ratio of apicobasal line tension to lateral surface tension. (E), (F), (G) Blue dots: experimental data, plotted as mean ± SD. Green line: theory (C, D) prediction for height, cell volume, and tissue area (see S1 Text). (Underlying data can be found at DOI: 10.5281/zenodo.1316912; for panel A at /Matejcic-et-al_2018/Data/F5A.csv and panel B at F5B_6D_S6.csv. The theoretical analysis for panels E-G can be found at /Matejcic-et-al_2018/Theory/Data analysis_essentials.nb.). hpf, hours post fertilization.

The redistribution of actin was not only linked to an increase in nuclear stacking but also coincided with the cell height increase observed after 36 hpf (Figs 2C and 5A).

As contraction and tension of the actin cytoskeleton can actively affect cell shape in many different developmental contexts [28–30], we asked whether actin redistribution was involved in cell elongation. To test this notion, we analyzed actin redistribution in cells by comparing the ratios of the lateral actin signal intensity to basal- and apical-most actin signal intensity throughout the proliferative phase (Fig 5B, S6 Fig). We found that, as development progressed, actin was depleted from the lateral cell cortex and concentrated at the basal and apical cell attachments, relative to its lateral distribution (Fig 5B, S6 Fig). If actin distribution was indeed involved in controlling tissue height, one simple explanation could be that the changing cell aspect ratio is dictated by a balance of forces between the line tensions in the apical and basal actin epithelial belt and the surface tension exerted by the actin cortex along the lateral cell interfaces (S4 Fig and S1 Text). The actin redistribution observed between 30 hpf and 42 hpf would then drive cell height elongation by favoring a contraction of the apical and basal perimeter at the cost of an expansion of the lateral surface area. We incorporated this possibility in our simplified description of tissue growth by considering a mechanical balance equation that gives the cell shape as a function of the lateral surface tension and the apicobasal line tensions (S4 Fig, S1 Text). We also proposed an overall description of tissue growth with a constant rate of volume growth and a constant rate of progenitor cell divisions. We further assumed that a transition occurs at approximately 35–36 hpf, at which progenitors start to differentiate, and actin redistributes from the lateral surfaces to apical and basal epithelial belts (Fig 5C and 5D). Taking into account the balance equations discussed earlier (S1 Text), this simplified description accounted for the main aspects of the geometrical changes seen for the tissue between 20 hpf and 48 hpf (Fig 5E–5G).

**Fig 6:**
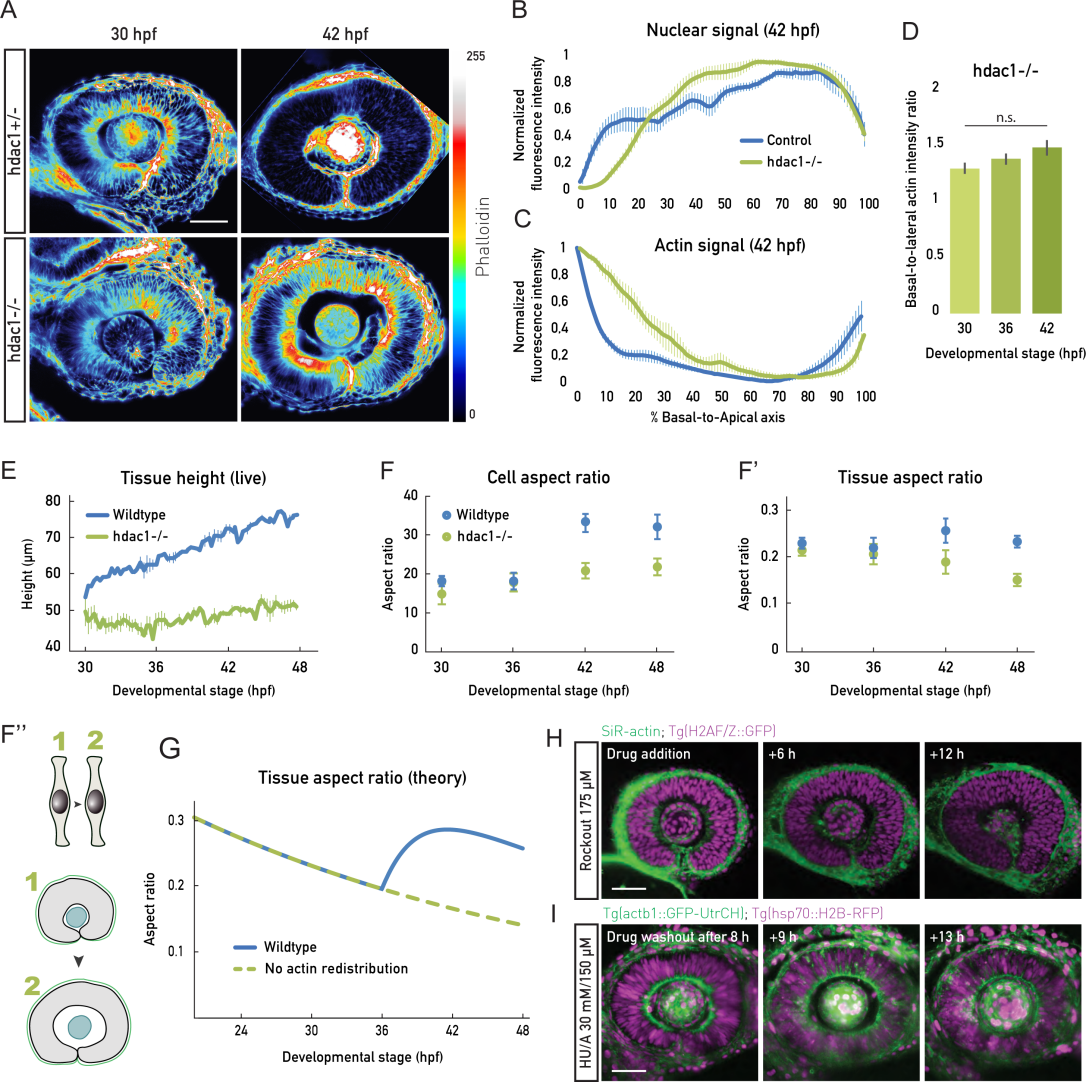
Actin redistribution enables cell elongation in concert with cell proliferation. (A) Phalloidin signal before (30 hpf) and after (42 hpf) actin redistribution in the heterozygous (hdac1+/−) controls and hdac1−/− samples. Basolateral actin accumulation does not redistribute in hdac1−/− samples (bottom panels). Lookup table indicates minimal and maximal phalloidin signal values. (B), (C) Normalized average intensity distributions of DRAQ5 (B) and phalloidin (C) signal in control (blue) and hdac1−/− (green) samples at 42 hpf. Values in each sample are normalized to minimum and maximum values. Data are shown as mean ± SEM; 3–9 samples/stage. (D) Basal-to-lateral phalloidin signal intensity ratios in hdac1−/− samples; 5 samples/stage. Mean ± SD. Mann-Whitney test, *p*-value 0.3095. (E) Tissue height measurements from live embryos (light sheet time lapses) for wild-type (blue) and hdac1−/− (green) samples related to S5 Movie). *N* = 2/condition. (F) Cell shape analyzed as aspect ratios from mean cell cross-sectional area and height in wild-type (blue) and hdac1−/− (green) samples; 3–10 samples/stage. Wild-type data same as in Fig 2E. (F’) Tissue shape analyzed as aspect ratios from mean tissue area and height in wild-type (blue) and hdac1−/− (green) samples; 3–10 samples/stage. Wild-type data same as in Fig 2E. (F”) Schematic representation of the unchanged cell aspect ratio during hdac1−/− retinal PSE growth and perturbed tissue shape. Related to S5 Movie. (G) Prediction of simplified theory for the tissue aspect ratio $\left(\mathrm{Height}/\sqrt{\mathrm{Area}}\right)$ in the model described in Fig 5C and 5D (blue line) and in a model in which the actomyosin does not redistribute from apicobasal junctions to the lateral cortex (green dotted line, see S1 Text for details, compare with Fig 6F’). (H) Rockout treatment (175 μM) abolishes the basolateral actin accumulation but is insufficient to induce cell elongation. Scale bar: 50 μm. Actin is labeled by SiR-actin (green), and nuclei by Tg(H2AF/Z::GFP) (magenta). (I) Proliferation is necessary for cell elongation. After HU/A treatment (30 mM HU + 150 μM A), cell proliferation is blocked, but cell height does not increase (see also S6 Fig). Actin is labeled by Tg(actb1::GFP-UtrCH) (green), and nuclei by Tg(b-actin::H2B-RFP) (magenta). (Underlying data can be found at DOI: 10.5281/zenodo.1316912; for panels B and C at /Matejcic-et-al_2018/Data/ F4CDE_6BC.csv, panel D at F5B_6D_S6.csv, panel E at F6E.csv, panels F and F’ at F6F.csv. The theoretical analysis for panel G can be found at /Matejcic-et-al_2018/Theory/Data analysis_essentials.nb.). A, aphidicolin; DRAQ5, deep red anthraquinone 5; hdac1, histone-deacetylase 1; hpf, hours post fertilization; HU, hydroxyurea; n.s., not significant; PSE, pseudostratified epithelium.

To experimentally test whether a link between actin redistribution, cell height increase, and uniform tissue growth existed, we used a histone-deacetylase 1 (hdac1) zebrafish mutant that undergoes continued proliferation and does not enter differentiation programs, because of the loss of Hdac1-dependent Wnt and Notch inhibition [31,32]. It was previously reported the hdac1−/− retinal PSE shows a perturbed tissue shape [31,32] (S5 Fig). Our live imaging confirmed that hdac1−/− mutants developed folds in the retinal PSE instead of the smooth tissue surface seen in controls (S5 Fig, S4 Movie). This shape disruption starts at stages at which differentiation would have begun in the wild-type retina (S5 Fig, S4 Movie). In previous studies, the emergence of this shape distortion was not explicitly explored, but it was reported that hdac1−/− mutants show disturbed actin organization [32]. We thus set out to test whether a lack of timely actin redistribution could be linked to the tissue shape disruption seen in this mutant. We imaged actin localization and cell height changes of control versus hdac1−/− neuroepithelia (Fig 6A and S5 Movie).

This revealed that in hdac1−/− embryos, actin accumulation along lateral interfaces lasted substantially longer than in controls (70 hpf versus 40 hpf, Fig 6A–6C). This was confirmed by the analysis of basal-to-lateral actin signal ratios, which, unlike the control, remained unchanged throughout development in the hdac1−/− embryos (Fig 6D).

With the basolateral actin accumulation maintained in hdac1−/− tissue, cells did not elongate past 55 μm (Fig 6E), a height value that is reached in controls at 30 hpf. As a consequence of hdac1−/− cells not changing their shape as control cells (Fig 6F), the hdac1−/− retinal PSE did not maintain its aspect ratio upon continued proliferation (Fig 6F’ and 6F”, S5 Movie), leading to tissue shape changes rather than cell shape changes. This indeed indicated a link between retinal PSE shape changes and actin rearrangements. In accordance with this finding, our simplified theory predicted that the lack of actin redistribution leads to a decrease in tissue aspect ratio, as seen in the hdac1−/− mutant (Fig 6G).

While retinal PSE shape was disturbed in the hdac1−/− neuroepithelium, the tissue nevertheless retained its pseudostratified characteristics, as shown by intact apical junctions and basal lamina (S5 Fig) and its mitoses occurring apically (S5 Fig). A similar sequence of tissue shape changes was seen when using an Hdac1 morpholino (S5 Fig) [31] or the Hdac1 inhibitor Trichostatin-A (S5 Fig). In all conditions, the end result of cell shape maintenance was the appearance of apical and basal epithelial folds (S5 Fig) [31,32].

Taken together, these data suggest that actin redistribution from lateral cellular interfaces to the apical and basal epithelial belts drives cell and thereby tissue elongation. When this reorganization does not occur, cells do not increase their height, leading to disturbed tissue shape.

### Actin redistribution allows for tissue height increase in concert with cell proliferation

We asked whether the depletion of actin from the lateral cellular interfaces is sufficient to drive cell elongation. To test this, we used Rockout as we showed that this depletes the basolateral actin accumulation prematurely (Fig 6H). As shown before (Fig 4F), this treatment led to nuclei redistributed along the complete apicobasal axis (Fig 6H). However, even after the actin accumulation was diminished and nuclei also inhabited basal positions, no increase in tissue height was observed (Fig 6H, S6 Movie). This counterintuitive result led us to further evaluate the effect of Rockout on overall tissue development. We noted that in the Rockout-treated embryos, cell division rate was reduced compared to controls (between 37 and 40 hpf, only 3 mitotic events were observed in the middle retinal section [S6 Movie], compared to 78 mitotic events in wild type in the same retinal region [S5 Movie]). This effect of Rockout on cell proliferation is most likely due to the effect the drug has on cytokinesis by inhibiting ROCK-mediated myosin light chain phosphorylation [33,34]. This consequently affects the tissue growth rate. Thus, actin disappearance from lateral cellular interfaces was necessary but not sufficient to trigger cell elongation. Instead, our experiments suggested that cell proliferation was another important factor to increase cell height in a growing PSE tissue. Indeed, the increase in cell height observed after 36 hpf coincided with increased proliferation due to shortened cell cycles (Fig 3D and 3E). To directly test whether proliferation was involved in cell elongation, we blocked proliferation by hydroxyurea/aphidicolin treatment at 30 hpf. In this condition, even after the lateral actin accumulation disappeared, the tissue height did not increase to the same amount as in controls (Fig 6I, S7 Fig). Using the same approach, we explored how proliferation inhibition influences tissue shape in the hdac1−/− mutant. Here, actin did not get redistributed from basolateral positions, and the tissue did not increase its height, as seen before (Fig 6A). Because of the lack of ongoing proliferation, however, no tissue buckling or shape disturbance was observed (S7 Fig).

Together, this set of experiments strongly argues that both the disappearance of actin from lateral cell interfaces and continued proliferation are required for the observed cell and tissue elongation. Therefore, these two factors together ensure tissue shape scaling during retinal PSE growth.

### Actin distribution reorganizes non-cell autonomously and is dependent on the ECM

**Fig 7:**
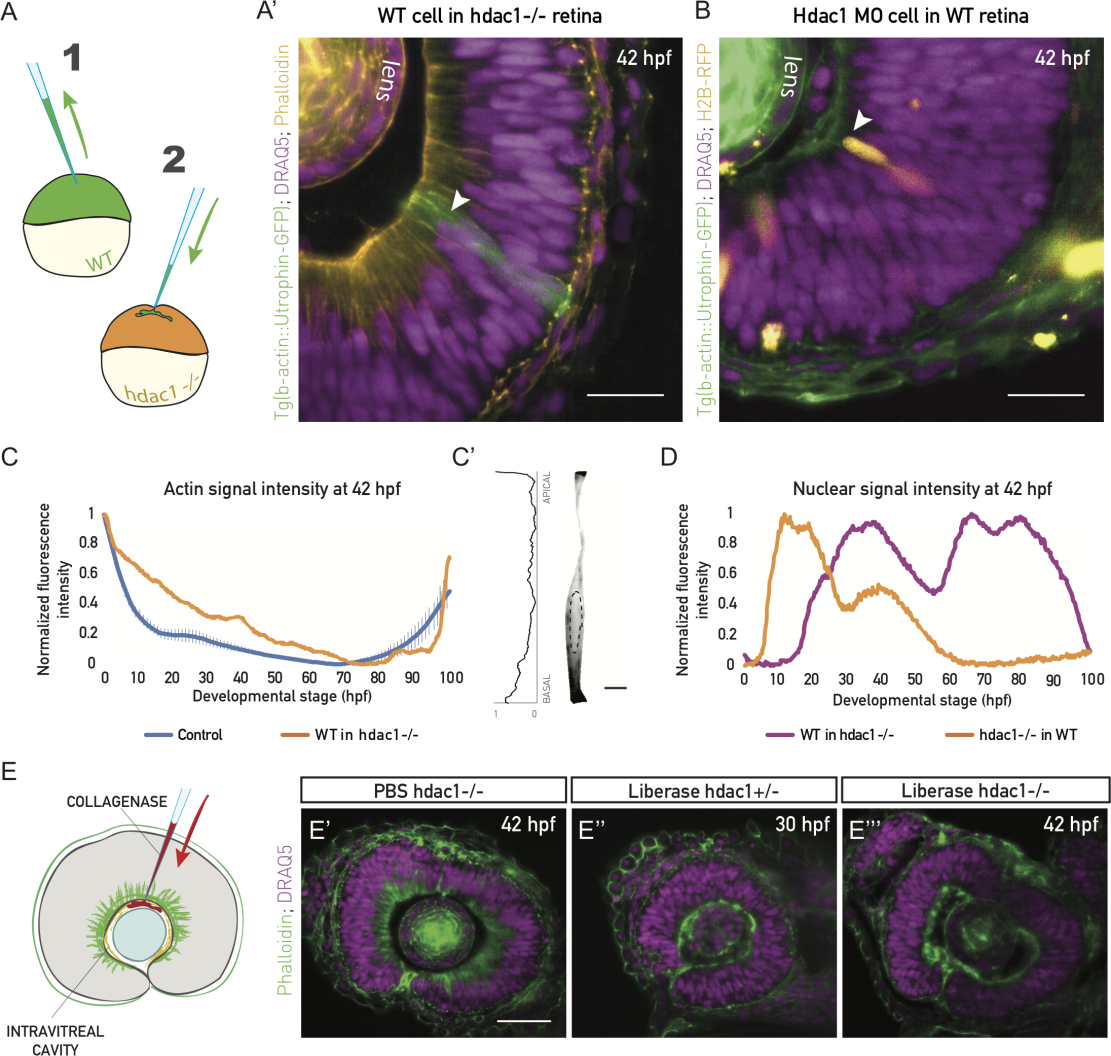
Actin reorganization and cell height increase are non-cell autonomous and ECM dependent. (A) Schematic of the cell transplantation experiment. Blastomeres from Tg(actb1::GFP-UtrCH) embryos (1) were transplanted into acceptor hdac1−/− embryos (2) between high and sphere stage. (A’) WT cells in hdac1−/− tissues do not redistribute basolateral actin, nor do they increase their height at 42 hpf (green cells, arrowhead). Actin is labeled with phalloidin (orange) and nuclei with DRAQ5 (magenta). *N* = 5 embryos. Scale bar: 50 μm. (B) Mosaic injection of hdac1 MO into Tg(actb1::GFP-UtrCH). Cells with hdac1 MO were co-injected with H2B-RFP mRNA. Their basal actin zone disappears at the same time as in the WT (orange cells, arrowhead). Actin is labeled with phalloidin (green) and nuclei with DRAQ5 (magenta). *N* = 5 embryos. Scale bar: 50 μm. (C) Normalized fluorescence intensity profiles of phalloidin signal along the apicobasal axis of the transplanted clone marked in (A’). Control plot is same data as Fig 4E. (C’) Phalloidin signal in a manually segmented transplanted PSE cell from sample in (A’), showing basolateral accumulation on the single-cell level. Dashed line outlines the cell nucleus. Scale bar: 5 μm. Left: Normalized basal-to-apical phalloidin signal intensity along the lateral cell membrane of cell on right. (D) Normalized fluorescence intensity profile of nuclear signal (H2B, orange line) along the apicobasal axis of the hdac1-deficient clone in (B) and nuclear signal (DRAQ5, magenta line) in the transplanted clone in (A’). (E) ECM integrity was perturbed by injecting 0.5 mg/ml of collagenase (Liberase) into the intravitreal cavity of the zebrafish eye. (E’) The basal actin accumulation in hdac1−/− tissues remains intact when PBS is injected as control. (E”) Collagenase abolishes the basal accumulation in 30 hpf control hdac1+/− embryos. (E”’) Collagenase abolishes the basal actin accumulation in 42 hpf hdac1−/− tissues. E’-E”’, actin is labeled with phalloidin (green), nuclei are labeled with DRAQ5 (magenta). (Underlying data for panels C and D can be found at DOI: 10.5281/zenodo.1316912; /Matejcic-et-al_2018/Data/F7CD.csv.). DRAQ5, deep red anthraquinone 5; ECM, extracellular matrix; hdac1, histone-deacetylase 1; hpf, hours post fertilization; MO, morpholino; PSE, pseudostratified epithelium; WT, wild type.

Live imaging of F-actin revealed that the disappearance of actin from the lateral interfaces occurred simultaneously in all progenitor cells throughout the retinal PSE (S7 Movie). This observation could be explained 2-fold: (i) a cell-intrinsic event is coordinated in all cells, or (ii) cells react to an extrinsic, tissue-wide signal. To differentiate between these two possibilities, we transplanted cells from control embryos into hdac1−/− embryos, in which the basolateral actin bias persisted (Fig 7A and 7A’).

Here, transplanted control cells preserved their actin distribution (Fig 7A’, 7C and 7C’) and did not increase their height, even at stages at which they would elongate in their native environment (Fig 7A’). Conversely, when hdac1 morpholino was injected into 64-cell stage control embryos to achieve a mosaic distribution of morphant cells, these morphant cells lost the lateral actin accumulation similarly to neighboring control cells (Fig 7B). In this context, nuclei of hdac1 morphant cells occupied basal positions (Fig 7B and 7D), and cells increased their height alongside their neighbors (Fig 7B). This confirmed that actin redistribution is a non-cell-autonomous event that coincides with cell height increase.

It has been previously shown that the ECM underlying growing tissues can actively influence their size and shape during organ development [35,36]. All retinal PSE cells are basally attached to the ECM of the basal lamina, which contains collagen and laminin as main components (S5 Fig) [37]. As forces mediated by cell–ECM attachments can affect actin polymerization and bundling in diverse epithelial cells [38,39], we tested whether cell–ECM interactions were involved in the tissue-wide non-cell-autonomous actin reorganization in the retinal PSE. We injected collagenase, an ECM-degrading metalloprotease, between the lens and the retinal neuroepithelium of hdac1+/− and hdac1−/− fish at 30 hpf and 42 hpf (Fig 7E). In the hdac1+/− control, as well as in the hdac1−/− tissues, collagenase indeed abolished the actin accumulation on lateral interfaces and led to the removal of the nuclear exclusion zone (Fig 7E” and 7E”’), which was not seen in PBS-injected controls (Fig 7E’). This suggested an active role of the ECM in actin organization. Thus, as the ECM is necessary for the distribution of lateral actin, it might also be driving its redistribution and, ultimately, the isotropic growth of the retinal PSE.

Together, these data show a strong relationship between actin redistribution and cell height increase, indicating that these parameters are mechanistically linked. Cell-level actin redistribution occurs independently of cellular identity but is most likely dependent on a change in the ECM to allow the maintenance of tissue shape during growth.

## Discussion

In this study, we used a combination of quantitative 3D tissue-wide analysis and long-term 3D live imaging to investigate tissue growth and shape. We demonstrated how such a large-scale, quantitative approach can be used to decompose growth of tissues into geometric cell and tissue-wide parameters to extract fundamental rules of growth during development.

**Fig 8:**
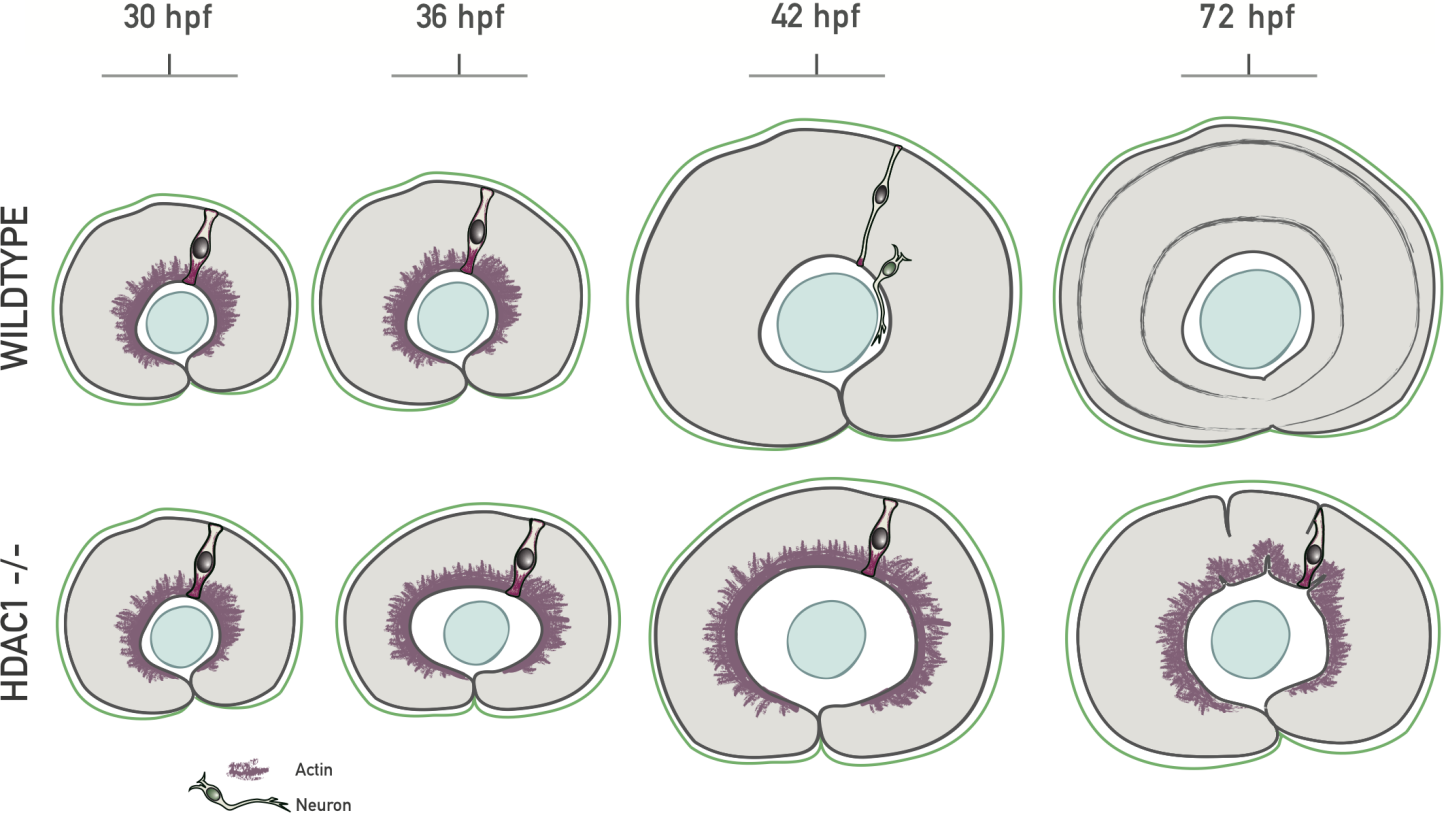
Model of basolateral actin redistribution enabling isotropic retinal growth. During growth of the wild-type zebrafish retinal PSE, a basolateral actin accumulation disappears tissue-wide between 36 hpf and 42 hpf, coinciding with an increase in cell height. In the hdac1−/− retinal PSE, the basolateral actin does not redistribute, and cells do not elongate past 55 μm. Such cytoskeletal redistribution mechanically enables cell elongation by changing the tension balance within the cells and, consecutively, results in maintenance of retinal tissue shape during proliferative growth. hdac1, histone-deacetylase 1; hpf, hours post fertilization; PSE, pseudostratified epithelium.

Our analysis on growth and shape showed that the retina growth is uniform and isotropic. We find that this uniform 3D growth is enabled by the redistribution of the actin cytoskeleton in concert with continued proliferation, allowing for timely apicobasal cell elongation ensuring tissue shape maintenance (summarized in Fig 8).

To understand the links between growth and shape in a developing PSE in 3D, we generated a quantitative dataset of the retinal neuroepithelium growth phase. This, together with a set of balance equations, allowed us to create an unprecedented characterization of growth. We reveal that geometric changes at the single-cell level are essential to maintain tissue shape. Single-cell shape changes were most prominent after 36 hpf, a stage at which tissue height increased.

In concurrence with this increase in tissue height, nuclei enter a previously unoccupied nuclear exclusion zone, leading increased nuclear stacking. We find that the loss of this nuclear exclusion zone, as well as cell height increase, is linked to a cellular redistribution of actin. When actin is maintained at lateral cell interfaces, cell elongation does not take place, and the scaling of tissue shape is consequently disturbed. These findings, together with a simplified theoretical description, led us to propose a model in which the lateral actin accumulation generates a mechanical force acting against apicobasal cell elongation. We postulate that the redistribution of actin leads to a change of distribution of forces generated in the cells, involved in driving apicobasal cell elongation. Because a similar redistribution of tension has been suggested to drive cell elongation in the developing *Drosophila* wing disc PSE [40], and due to the fact that all PSE generally thicken during growth, we propose that such a mechanism might be evolutionarily conserved in other developing PSE tissues of intermediate length. How exactly height increase results from the observed actin redistribution will need to be further investigated.

Basolateral actin accumulation depends on an intact underlying ECM, as we found that disturbing ECM composition results in actin redistribution. This, together with the fact that actin redistribution occurs non-cell autonomously, supports the idea that ECM rearrangements or cell–ECM attachments act as upstream modulators of actin cellular organization in the retinal PSE. In line with this assumption, distinct ECM components have been shown to play an instructive role in regulated proliferation in the vertebrate central nervous system (CNS) as well as retinal development [41,42].

Upstream drivers for the ECM-mediated actin redistribution here could be, for example, the Wnt or the Notch signaling pathway, as Hdac1 acts as inhibitor of Wnt and Notch, and both molecules have been implicated in modulating cell–ECM force transmission [43–45]. Overall, our finding showcases an example of a developmental change in which the ECM can actively influence tissue size and shape during growth, as also seen, for example, in the *Drosophila* wing disc PSE [35] or the developing mouse salivary gland [36].

Our results also demonstrate that tissue growth needs to be tightly coordinated to cell shape changes in time and space for successful tissue development. We show that cell height increase occurs in concert with the neighboring tissue independently of how the cell would behave in its native environment. In contrast, retinal neuronal differentiation is most likely a cell-autonomous program, as we observed that after washing out the hdac1 inhibitor Trichostatin-A, cells in the already folded retinal tissue regained the potential to differentiate and generate neuronal layers, in spite of the tissue’s perturbed shape (S5 Fig). Thus, it is likely that differentiation is uncoupled from tissue shape. This lends further weight to the conclusion that precise coordination of cell-intrinsic and tissue-wide parameters is essential to generate the fully functional, differentiated tissue of correct size and shape.

We found that in the retinal PSE, apical surface availability does not pose a constraint on growth during the proliferative phase, as was suggested for other PSE [15,17,25]. While it is possible that the retinal PSE is an exception due to its curved shape, a careful analysis of factors contributing to tissue growth in other PSE, similar to our study here, would allow testing of this old hypothesis [17] in other contexts.

As retinal cells need to change their geometry while the tissue grows, our study exemplifies that tissue shape maintenance during growth is not a default state but requires active preservation [46]. We found that shape scaling of the retina depends on altering active forces in the cell that control the cell shape. Such a dynamic force change is also seen in other contexts such as germ band elongation in *Drosophila*, in which actin redistributes along the apical plane of the epithelium [4]. In the example presented here, however, we propose that cellular tension redistribution acts in 3D, along the lateral cell surfaces, as well as along apical and basal cell perimeters, to ensure scaling of shape during growth, rather than guiding changes in tissue shape.

Overall, we here elaborate on the important question of how organs coordinate growth with scaling of shape during development. We believe that our quantitative dataset can constitute a reference point when assessing remaining questions of growth in diverse developing PSE. It has recently become more feasible to test such questions in developing embryos in vivo due to new and improving imaging techniques [47]. Quantitative studies as presented here will need to be extended in the future, using more model tissues and organisms. A future challenge will be to expand investigations to growth phenomena within *ex vivo* tissues and organoid systems, including human organoids, which often emerge from PSE, as seen for retinal and cerebral organoids [48–51]. In-depth 3D studies of growth and shape like presented here will allow to compare growth phenomena of *ex vivo* and organoid cultures to in vivo growth. Such comparisons will deepen our understanding of developmental programs in model organisms and humans.

## Materials and methods

### Ethics statement

The experiments were approved and licensed by the local animal ethics committee (Landesdirektion Sachsen, Germany; license no. DD24-5131/354/11) and carried out in accordance with the European Communities Council Directive of 22 September 2010, as well as the German Animal Welfare Act.

### Zebrafish husbandry

Zebrafish were maintained and bred at 26.5°C. Zebrafish embryos were raised at 21°C, 28.5°C, or 32°C and staged in hpf according to [52]. E3 medium was exchanged daily; 0.003% 1-phenyl-2-thiourea (PTU) was added to the embryo E3 medium from 8 ± 1 hpf onwards and renewed daily to prevent pigmentation. Embryos were anesthetized in 0.04% tricane methanesulfonate (MS-222; Sigma-Aldrich) prior to sorting for fluorescent signal (>36 hpf), imaging, intravitreal cavity injections, brain ventricle injection, or retinal dissection.

### Transgenic and mutant lines

See S1 Table for a list of genetically modified lines used in this study.

### Hdac1 mutant line

Hdac1 t24411 mutants containing a single point mutation in the hdac1 gene were obtained from the Zebrafish International Resource Center (ZIRC). Incrossed, homozygous fish (hdac1−/−) until 3 dpf were used in experiments.

### Blastomere transplantation

Donor embryos (Tg[actb1::GFP-UtrCH]) and acceptor embryos at stages high to sphere were dechorionated in pronase, and cells from the animal pole were transplanted into the acceptor hdac1+/−, −/− embryos. Transplanted embryos were kept on agarose for about 3–5 h and then transferred onto glass dishes. The E3 medium was supplemented with antibiotics (100 U of penicillin and streptomycin, Thermo Fisher Scientific). Homozygous mutants were identified, fixed, and stained after 30 hpf.

### Morpholino injections

Morpholinos were acquired from Gene Tools.

Hdac1 morpholino was used to knock down Hdac1 mosaically, or in all cells. p53 morpholino was added to the morpholino injection mix to alleviate negative effects associated with injection and knockdown. All morpholino mixes were injected into the yolk of 1- to 2-cell stage embryos. See S2 Table for used morpholinos.

### mRNA and plasmid injections

To label all cells, mRNA was injected into the yolk or cell of 1-cell stage embryos (100 pg/embryo). The injection volume was 1–2 nl. mRNA was synthesized using the Ambion mMessage mMachine kit. To label cells mosaically, mRNA was injected into single cells of 32- to 128-cell stage embryos (50 pg/cell in 0.5 nl injection volume). Plasmid DNA was injected into the cell of 1-cell stage embryos (5–15 pg/embryo in 1–2 nl).

### Intravitreal cavity collagenase injections

To perturb the ECM locally, collagenase (Liberase, Sigma Aldrich), an ECM-degrading metalloprotease, was injected into the intravitreal cavity (between the lens and the retina). Then, 0.5 mg/ml Liberase in a volume to fill the cavity (2–3 nl) was injected in 36 hpf or 42 hpf hdac1−/− embryos and 30 hpf control embryos. Injected fish were incubated in a water bath at 37°C to activate the enzyme for 30 min. Afterwards, they were fixed and stained with phalloidin.

### Heat shock of embryos

Embryos injected with plasmids containing Hsp70::H2B-RFP, Hsp70-EGFP-PCNA, or transgenic embryos (Tg[Hsp70::H2B-RFP]) were heat-shocked at least 1.5 h prior to imaging. Embryos injected with Hsp70::H2B-RFP or Hsp70-EGFP-PCNA plasmid were heat-shocked for 10 min at 37°C. Tg(Hsp70::H2B-RFP) embryos were heat-shocked for 20 min at 38°C.

### Drug treatments

The minimal effective concentrations of inhibitors were optimized based on dilution series. All inhibitors were dissolved in DMSO, except for hydroxyurea, which was dissolved in water. Dechorionated embryos were treated by incubation in the inhibitor-E3 medium at specific concentrations, either in plastic multiwell plates or directly in a light sheet microscope chamber (Rockout, Trichostatin-A). All treatments started after 24 hpf (Rockout in wild type always before 42 hpf) and lasted from 3 h to 2 d (Trichostatin-A), with most treatments lasting overnight (about 15 h). See S3 Table for the list of used chemical inhibitors and their concentrations.

### Immunostaining

Whole-mount immunostainings were performed on pronase-dechorionated embryos, fixed in 4% paraformaldehyde (Sigma) in PBS, at +4°C overnight. Washes were performed in PBS-T (PBS with 0.2% or 0.8% Triton X-100). Embryos were permeabilized in 1% Trypsin and blocked in 10% normal goat serum (NGS) or blocking buffer [53]. Embryos were shaken in primary antibodies for 64 h at +4°C.

#### Primary antibodies

The phosphorylated histone H3 (PH3) antibody (Abcam; ab10543, RRID:AB 2295065) was used at 1:500 to label chromatin of mitotic cells. Laminin α1 antibody (Sigma-Aldrich; L9393, RRID:AB_477163) and Collagen IV antibody (Abcam; ab6586, RRID:AB_305584) were both used at 1:100 to label the basal lamina.

#### Secondary antibodies

Secondary antibodies with conjugated fluorophores Alexa Fluor 405, Alexa Fluor 488, Alexa Fluor 568, and Alexa Fluor 647 (Thermo Fisher Scientific) were used at 1:500 or 1:1,000.

#### Fluorophore-conjugated compounds

To label all nuclei, DAPI (1:5,000 or 1:2,500) and DRAQ5 (1:2,500, Thermo Fisher Scientific; 62251) were used. Phalloidin-TRITC (1:50, Life Technologies; R415) and phalloidin-Alexa Fluor 488 (1:50, Life Technologies; A12379) were used to label F-actin.

### Cell dissociation and flow cytometry

To obtain relative numbers of differentiating cells in proportion to all retinal PSE cells, Tg(SoFa) retinas [23] were dissected, dissociated, and analyzed by FACS.

#### Retinal dissection and dissociation

Dissection was performed on Sylgard-coated 10 cm plastic dishes at room temperature in PBS. Eyes were dissected from live 36 hpf, 42 hpf, and 48 hpf Tg(SoFa) and 48 hpf wild-type anesthetized embryos using forceps, syringe needles, and/or a 5 mm surgical stab knife. The pigment epithelium and lens were removed. In total, 20 retinas/stage were transferred to a glass FACS tube. Cells were dissociated mechanically and immediately analyzed by FACS.

#### FACS sorting

Single-cell suspensions were analyzed using the FACSAria device. Wild-type cells were used to normalize the signal to background autofluorescence. Populations were defined based on forward- and side-scatter signals in PE-A, FITC, and DAPI channels. Data were plotted and processed using the FACSDiva software. The resulting scatterplots were analyzed as described previously for the SoFa line [23] in order to obtain relative values for different neuronal subtypes. Total numbers of cells in the retina at different developmental stages were used to calculate average absolute numbers of differentiated cells from the relative values.

### Image acquisition

#### Confocal laser point-scanning microscopy

Dechorionated, fixed, and immunostained embryos were mounted in 1% low-melting-point agarose in glass-bottom dishes (35 mm, 14 mm microwell, MatTek). The agarose dome was covered with PBS or E3. Imaging was performed on Zeiss LSM 510 or 710 confocal microscopes (Carl Zeiss Microscopy) using a 40×/1.2 NA water immersion objective at room temperature. The imaging system was operated by the ZEN 2011 (black) software.

#### Light sheet microscopy

Dechorionated live or fixed embryos were mounted in glass capillaries in 0.6% or 1% low-melting-point agarose. Imaging was performed on a Zeiss Light sheet Z.1 microscope (Carl Zeiss Microscopy) with a Zeiss Plan-Apochromat 20× or 40× water-dipping objective (NA 1.0) at 28.5°C. For drug treatment experiments, the chamber was filled with the drug-supplemented medium. For fixed-sample imaging, embryos were mounted in 1% or 0.6% agarose, immersed in PBS, and imaged at room temperature. Z-stacks spanning the entire eye were recorded with 0.5–1 μm optical sectioning. For live imaging, z-stacks were recorded every 5–30 min. The system was operated by the ZEN black software.

#### Image analysis

Minimal image preprocessing was implemented prior to image analysis. Processing consisted of extracting image subsets, bleach correction, and/or background subtraction using Fiji [54]. XY-drift was corrected using a Fiji plugin (Manual Registration, Scientific computing facility, MPI-CBG). After image analysis in Imaris 8 (BitPlane) or Fiji, data were analyzed and plotted using MATLAB or Microsoft Excel. Statistical analysis was performed using the Prism software (version 6.0c for Mac OS; GraphPad Software).

#### Growth analysis

The majority of image analyses were done on the entire retinal tissue in 3D, using Imaris 8. This included tissue and cell volume, total tissue area, tissue height, cell and mitotic cell position, and count analyses. Raw data were segmented by manually outlining the retinal and lens tissues. Overall, 30–50 z-sections were outlined in each sample to span the retinal tissue, and 10 samples/stage, in 6 stages, were analyzed as part of the wild-type growth characterization. The segmented rendered surface was used to mask the raw data and create a subset on which further measurements were performed.

Total cell numbers were analyzed using the Imaris Spot detection tool to detect individual nuclei on the masked tissue-wide retinal stacks from the DRAQ5/DAPI nuclear signal. The automatic Imaris detection was first validated manually, by examining individual z-planes using the Oblique slicer tool and a combination of 2 Clipping plane tools to isolate and examine a specific tissue region. Automatic settings (thresholds) were manually adjusted for each sample to yield the best possible detection. To validate the total cell count, cell volumes were estimated from mitotic cell volumes (see above). The total tissue volume was divided by these cell volumes, in order to obtain the average volume occupied by a single cell (i.e., cell volume). Cell volumes obtained in this way were in very good agreement to the values obtained by the automatic count (S1 Fig), validating the cell count analysis.

For fixed samples of wild-type and hdac1−/−, thickness was measured using Imaris in 3D in the central z-slices of the nasal and temporal region of the retina and in the proximal region (from behind the lens to the deepest plane of the tissue). The length of the apicobasal tissue axis was measured. To correct for the lower thickness in the ciliary marginal zone (CMZ) around the optic fissure, all tissue thickness values were decreased by 5%, a value obtained by measuring average CMZ thickness in 5 exemplary samples. To manually measure thickness in live images of wild-type and hdac1−/− fish, the Fiji Line tool was used. The apicobasal tissue axis at the nasal region of the retina was taken as a representative region and measured in the mid-retinal plane at every time point.

Apical tissue surface area of the retinal PSE was analyzed and visualized in 3D using a custom Fiji tool (Volume Manager, by Robert Haase, Scientific computing facility, MPI-CBG). Volume Manager is available from the Fiji SCF-MPI-CBG update site. Apical surfaces were manually outlined through the tissue-wide retinal image stack. The basal tissue area was calculated by subtracting the apical tissue surface area from the total tissue surface area, obtained from the Imaris 3D manual segmentation. Average apical and basal cross-sectional cell areas (surfaces of apical and basal cell attachments) were calculated by dividing the apical and basal tissue area by the total number of cells, respectively, and by calculating the arithmetic mean of thus obtained apical and basal cell attachments.

Cell size was estimated using diameters of rounded, mitotic cells. Diameters of 15–20 mitotic cells/retina (5–10 samples/stage) were measured using the Imaris Measuring tool at the widest point. Assuming that mitotic cells are nearly spherical, cell volumes were calculated as sphere volumes. To assess how well volumes of mitotic cells represent interphase cells, several interphase cell volumes were manually segmented from images with extremely sparse mosaic labeling of cells with GFP-Ras mRNA (cell membrane marker). This analysis indicated that mitotic cell volumes are, on average, 10% larger than interphase cell volumes, and the mitotic cell volume measurements were adjusted accordingly to better reflect interphase cell volumes.

#### Mitotic density distribution

To assess the distribution and the density of mitoses at the apical surface in different stages, mitotic cell positions were detected and projected onto a 2D density heatmap, using a custom MATLAB tool, “PSE MitoNuc” (Benoit Lombardot, Scientific computing facility, MPI-CBG). First, all PH3+ mitotic cells in the masked retinal stack were detected in Imaris using the Spot detection tool. Then, 3D Cartesian coordinates of all detected spots were exported from Imaris and analyzed by the “PSE MitoNuc” tool. Briefly, the 3D mitotic coordinates were first transformed into spherical coordinates and then projected onto a 2D surface using an existing density-preserving, azimuthal projection tool. To use the “PSE MitoNuc” tool, refer to the readme file of “PSE_MitoNuc” (DOI: 10.5281/zenodo.1316912). This method was validated on terminal neurogenic divisions (Ath5+), and the typical nonuniform distribution of divisions (naso-temporal wave) was clearly detected here (S2 Fig).

#### Division orientation

Division orientations were analyzed manually in Fiji from images of cells labeled with Tg(actb1::HRAS-EGFP) (membrane) and in fixed samples with the mitotic marker antibody phospho-histone 3 (PH3). A part of the curved apical surface was imaged using the light sheet microscope. Data were bleach-corrected, and background was subtracted. For each observed anaphase cell, a line was drawn perpendicular to the division plane, and the angle was measured with respect to the temporal-nasal tissue axis (horizontal image axis). Data were plotted as a polar histogram in MATLAB.

#### Cell cycle analysis

Mosaically labeled cells in long (>15 h) time-lapse light sheet datasets, with time resolution of 5 min, were manually tracked in 3D in ZEN and Fiji. The majority of cell cycle data come from cells labeled with nuclear markers Hsp70::H2B-RFP [20] or Hsp70::EGFP-PCNA [21]. For total cell cycle analysis, time of chromosome segregation for the first, second, and, in wherever possible, third division was recorded. For the analysis of mitosis length, end points of mitosis were recorded as the point of cell rounding and the point of chromosome segregation. These cell cycle data for 254 cells from 20 embryos were analyzed, binned into discreet stages, and plotted in MATLAB.

#### Apical mitotic occupancy

Average cross-sectional surface areas of mitotic cells were calculated as circle surfaces from diameters of mitotic cells measured in fixed samples (20–40 cells/stage, 3–7 embryos/stage). The largest diameter was used, accounting for the widest cross-section of the mitotic cell and assuming that the apical surface of the tissue crossed the mitotic cells at this position. This number was multiplied by the number of mitotic cells in each stage to obtain the total tissue apical area occupied by mitotic cells. This area was divided by the total 3D tissue-wide apical surface area of the tissue to obtain the fraction of the apical surface occupied by mitotic cells in each stage.

#### Nuclear packing

The number of nuclei stacked in layers in the nuclear zone was calculated by dividing the average height of the nuclear zone of the tissue measured from nuclear stainings by the average length of the long (apicobasal) axis of the cell nuclei (30 nuclei/stage, 2 embryos/stage). The number of stacked nuclei along the entire apicobasal tissue axis was calculated at every stage by dividing the tissue height by the long axis of the nuclei. In addition to this, the number of layers was counted manually. These values were in very good agreement to the calculated values for the nuclear zone.

To calculate the number of nuclei that can be accommodated under a mitotic cell in each developmental stage, the volume of the truncated conical unit under the mitotic cell was calculated as the frustum volume

$$V=\frac{\pi }{3}\times h(R^{2}+r^{2}+R\times r),$$ (1)

where h is the mean cell (tissue) height, R the mean radius of a mitotic cell (large frustum base, see paragraph on apical mitotic occupancy), and r the small base of the frustum, calculated from the large base divided by the ratio of apical to basal tissue area. Mitotic cell cross-section and basal attachments are assumed to be circular. The nuclei that fit under the rounded mitotic cell are assumed to enter mitosis at the same position, once the apical surface is vacant. Therefore, the larger the mitotic cell, the higher the theoretical maximal number of nuclei under it. The average cell volume density (cells/μm^3^) in the tissue was calculated by dividing the total number of cells by the tissue volume. The number of nuclei (cells) within the mitotic frustum was calculated by multiplying the cell volume density by the volume of the frustum. This makes the simplifying assumption that the cell volume density in the frustum is equal to the cell volume density in the whole tissue.

Due to the apical localization of mitoses in the PSE, the average cell cycle time T_CC_, excluding mitosis (i.e., time during which a nucleus is not rounded at the apical surface), needs to be longer or equal to the duration of mitosis T_M_ (time during which a cell is occupying the apical surface) of all cells in the frustum unit, N [17].

$$(T_{CC}-T_{M})\geq T_{M}\times N$$ (2)

From Eq 2, the maximal number of cells in the frustum unit depends on the cell cycle parameters and is equal to

$$N=\frac{T_{CC}-T_{M}}{T_{M}}.$$ (3)

#### Actin intensity distribution

The average intensity distribution of phalloidin and DRAQ5 signal along the apicobasal axis of the PSE cells was measured as described in [2], using a custom Python script (Benoit Lombardot and Robert Haase; “IntensityDist.py” at DOI: 10.5281/zenodo.1316912). The region of interest was defined as a 10 μm × 10 μm × thickness cuboid (3–9 samples/stage). An average intensity value for this region was calculated for each point along the apicobasal axis. The axis length was normalized to 100. To compare different samples, the average intensities were normalized to the minimal and the maximal intensity value along the axis.

To obtain the ratios of actin signal intensity, the apical, basal, and lateral portions of the tissue were manually outlined using the Fiji line tool in the central section of the retina for 5 samples/stage. Apical and basal surfaces were outlined with 10 pt thick lines. Lateral regions were selected using 200–350 pt thick lines in the nasal, central, and temporal part separately. Intensity profiles were processed to extract peaks of signal, assumed to represent the cortical signal, using the MATLAB *findpeaks* function.

## Supporting information

S1 Table Zebrafish transgenic lines and mutant used in this study. (PDF)

S2 Table Morpholinos used in this study. (PDF)

S3 Table Chemical inhibitors/drugs used in this study. (PDF)

S1 Text Supplementary theory notes. (PDF)

S1 Fig Validation of cell count. (A) The cell count (red) was validated using mitotic cell volumes by dividing the average tissue volume by 90% of mitotic cell average volume for each stage (see Materials and methods). Data are plotted as stage mean ± SD. *n* = 10 samples/stage for all. (Underlying data can be found at DOI: 10.5281/zenodo.1316912; /Matejcic-et-al_2018/Data/F1_2_3D_S12BD34.csv.).
(TIFF)

S2 Fig Retinal mitoses and differentiation. (A) Heatmaps of Ath5+ (neurogenic) mitotic divisions at 36 hpf and 42 hpf. This validation shows that nonuniform cell divisions are detectable using the method in Fig 3A. Neurogenic divisions are spatially nonuniform, progressing through the tissue as a naso-temporal wave. (B) Duration of mitosis does not change over development. Cells labeled mosaically with Hsp70::H2B-RFP or Hsp70::EGFP-PCNA were tracked in light sheet time lapses at 5 min time resolution. Data were binned as developmental stage +/− 3 h. *N* = 197 cells from 20 embryos (24 hpf *N* = 20; 30 hpf *N* = 56; 36 hpf *N* = 57; 42 hpf *N* = 53; 48 hpf *N* = 11). (C) Retinal neurogenesis. Average number of neuronal subtypes, as analyzed by FACS from pooled dissected Tg(SoFa) retinal samples. *N* = 20 retinas/stage. Data were normalized to wild-type background fluorescence. (D) Cell density was calculated by dividing the number of cells by total tissue volume. *N* = 10 samples/stage. (Underlying data can be found at DOI: 10.5281/zenodo.1316912; for panels B and D at /Matejcic-et-al_2018/Data/F1_2_3D_S12BD34.csv, panel C at S2C.xlsx.). Ath5, atonal homolog 5; FACS, fluorescence-activated cell sorting; hpf, hours post fertilization.
(TIFF)

S3 Fig Mitotic cells at the apical surface of the retinal PSE. (A) Left: Schematic representation of PSE tissue architecture, with apical mitoses, migrating nuclei (arrows), and the mitotic frustum. The mitotic frustum is depicted as a conical unit below the rounded mitotic cell. We assume that all interphase nuclei in a single mitotic frustum (gray ellipses) undergo mitosis at the same position at the apical surface (gray). Middle: Schematic top view onto the apical surface cross-section (gray plane) marked in the left schematic. Interphase cells’ apical attachments are not shown. Right: Apical surface of the retinal PSE at 35 hpf, with cross-sections of mitotic and interphase cells. Cell membranes are labeled with Tg(actb1:HRAS-EGFP). Frame from Video 2. M: mitotic cells. Scale bar: 10 μm. (B) Fraction of the apical tissue surface area occupied by mitotic cells; 10 samples/stage. Related to Fig 3G. (Underlying data can be found at DOI: 10.5281/zenodo.1316912; /Matejcic-et-al_2018/Data/F1_2_3D_S12BD34.csv.). hpf, hours post fertilization; PSE, pseudostratified epithelium.
(TIFF)

S4 Fig Simplified description of zebrafish retina growth between 20 hpf and 48 hpf. (A) Schematic of division and differentiation rules considered in the simplified description of retina growth. For simplicity, we consider 2 cell populations—progenitors (white) and neurons, or committed precursors (gray). Progenitors divide with a constant rate *k*. Cell division give rise to 2 progenitors with probability *p*, 2 neurons/committed precursors with probability (1−*p*)*α*, 1 progenitor, and 1 neuron/committed precursor with probability (1−*p*)(1−*α*). Here, we assume that only symmetric differentiation events occur, such that *α* = 1. (B) Schematic of cell and tissue shape geometry. Cells are represented by truncated cones with apical and basal line tensions *Λ*_*a*_ and *Λ*_*b*_, and lateral surface tension T_*l*_. The apical and basal tissue surface area is obtained in the simplified description by multiplying the cellular apical and basal area by the number of cells. (C) Experimental data for the cell cycle time, tissue volume and ratio of apical to basal surface area as a function of time, and fit to a constant average value (cell cycle time and ratio of areas) or to an exponential (tissue volume). (D) Comparison between experimentally measured cumulative logarithmic rates of change of tissue volume, number of cells, cell volume, tissue area and tissue height, and cumulative logarithmic rates obtained from the simplified description. (Underlying data can be found at DOI: 10.5281/zenodo.1316912; /Matejcic-et-al_2018/Data/F1_2_3D_S12BD34.csv. The theoretical analysis can be found at /Matejcic-et-al_2018/Theory/Data analysis_essentials.nb.). hpf, hours post fertilization.
(TIFF)

S5 Fig Hdac1−/− characterization. (A) Phalloidin (green) and PH3 antibody (magenta) staining of F-actin and mitotic cells in about 70 hpf hdac1−/− retinal PSE. Apical mitoses, junctional belts, and basal actin accumulation are preserved in the folded tissue. (B) Laminin-alpha1 and Collagen IV (B’) antibody staining of wild-type and hdac1−/− retinal tissues around 48 hpf. An intact basal lamina underlies the basal surface of the hdac1−/− retinal PSE. (C) Tissue thickness does not increase in Hdac1 morpholino-injected or TSA-treated retinal PSE (42 hpf). (D) Phalloidin staining of approximately 72 hpf hdac1−/−. Epithelial folds form throughout the retinal PSE. (E) Tg(Ath5::GFP) retinas (red) treated with TSA at 24 hpf for 24 h and stained with phalloidin (green) and DRAQ5 (blue). The medium was replaced after 24 h. The PSE differentiates by 70 hpf despite tissue shape perturbed by folds. Consequently, neuronal layers are perturbed, as well. Scale bars: 50 μm for all images. DRAQ5, deep red anthraquinone; Hdac1, histone-deacetylase 1; hpf, hours post fertilization; PH3, phosphorylated histone H3; PSE, pseudostratified epithelium; TSA, Trichostatin-A.
(TIFF)

S6 Fig Tissue-wide actin intensity ratios. (A) Ratios of apical-to-lateral phalloidin signal intensity in control hdac1+/− tissue over development; 5 samples/stage. Mean ± SD. Mann-Whitney test, *p*-value 0.0317. (B) Ratios of apical-to-lateral phalloidin signal intensity in hdac1−/− tissues over development; 5 samples/ stage. Mean ± SD. Mann-Whitney test, *p*-value 0.0159. (C) Ratios of basal-to-lateral phalloidin signal intensity at 42 hpf in control and hdac1−/− tissues (*N* = 5) and in hdac1−/− tissue treated with 150 μM Rockout (*N* = 6). Rockout treatment abolishes the basolateral actin accumulation in hdac1−/− and restores the basal-to-lateral actin ratio to control values. Mean ± SD. Mann-Whitney test, control versus hdac1−/− *p*-value 0.0317; hdac1−/− versus hdac1−/− Rockout *p*-value 0.0043; control versus hdac1−/− Rockout *p*-value 0.4127. (Underlying data can be found at DOI: 10.5281/zenodo.1316912; /Matejcic-et-al_2018/Data/F5B_6D_S6.csv.). hdac1, histone-deacetylase 1; hpf, hours post fertilization.
(TIFF)

S7 Fig Proliferation is necessary for cell height increase. (A) Cell height of live (light sheet time lapses) Rockout-treated wild-type retinal PSE (gray). Sample was incubated in 175 μM Rockout for the entirety of the imaging session. Around 37 hpf, the basolateral actin accumulation was abolished, nuclei filled the basal positions, and proliferation stopped. Related to S6 Movie. (B) Cell height of live (light sheet time lapses) wild-type retinal PSE, treated with a combination of cell cycle inhibitors HU/A (red); 30 mM HU and 150 μM of A were added at the beginning of the movie (30 hpf). Proliferation stopped 3 h later (red arrowhead). Cell height did not increase further after cells cycle was blocked. Control plots (blue) in (A) and (B) are data from Fig 6E. (C) Top panels: Retinas of mutant fish treated with DMSO fold and proliferate normally (PH3 staining). Bottom panels: Mutant retinas treated with HU/A do not fold when their proliferation is inhibited but do maintain the basolateral actin accumulation. Scale bar: 50 μm. (Underlying data can be found at DOI: 10.5281/zenodo.1316912; /Matejcic-et-al_2018/Data/FS7A.csv and FS7B.csv.). A, aphidicolin; hpf, hours post fertilization; HU, hydroxyurea; PH3, phosphorylated histone H3; PSE, pseudostratified epithelium.
(TIFF)

S1 Movie 3D growth analysis workflow. Example of retinal neuroepithelial segmentation and analysis of mitotic density. All nuclei are marked with DAPI (blue). Mitotic nuclei are labeled with PH3 antibody (white, later magenta spheres). Spot tool in Imaris was used to automatically detect mitotic nuclei at apical surface (magenta spheres). Surface tool was used in Imaris to manually segment retinal neuroepithelial and lens outlines (gray and light blue). Scale bar = 50 μm. PH3, phosphorylated histone H3.
(MOV)

S2 Movie Analysis of cell division angles at apical surface. Time lapse of Tg(actb1::HRAS-EGFP) (gray) embryos at 35 hpf. View toward apical side of epithelium. Angles of dividing cells were analyzed perpendicular to division plane, using line tool in FIJI. Time in h:min. Scale bar = 10 μm. Related to Fig 3C.
(MOV)

S3 Movie Cell cycle analysis in retinal PSE. Time-lapse imaging of an embryo injected with Hsp70::H2B-RFP (gray) DNA. Red dot marks nucleus followed over multiple cell cycles. Cell cycle length was measured as time between subsequent cell divisions. Time in h:min. Scale bar = 10 μm. Related to Fig 3E. PSE, pseudostratified epithelium.
(MOV)

S4 Movie Shape perturbation of hdac1−/− retinal PSE. Time-lapse imaging of an hdac1−/− embryo injected with Ras-GFP RNA for uniform labeling (black). Time in h:min. Scale bar = 50 μm. Related to Fig 6. GFP, green fluorescent protein; hdac1, histone-deacetylase 1; PSE, pseudostratified epithelium.
(MOV)

S5 Movie Shape changes over growth in control versus hdac1−/− retinal PSE. Time-lapse imaging of a control and an hdac1−/− embryo injected with Ras-GFP RNA for uniform labeling (gray). Time in h:min. Scale bar = 50 μm. Related to Fig 6. GFP, green fluorescent protein; hdac1, histone-deacetylase 1; PSE, pseudostratified epithelium.
(MOV)

S6 Movie Growth of the Rockout-treated retinal PSE. Time-lapse imaging of a control Tg(H2AF/Z::GFP) embryo treated with 175 μM Rockout and injected with SiR-actin for uniform actin labeling. The Rockout treatment started at the start of the movie and continued for the entire duration of imaging. Time in h:min. Scale bar = 50 μm. Related to Fig 6. PSE, pseudostratified epithelium.
(MOV)

S7 Movie Tissue-wide lateral actin redistribution during retinal PSE growth. Time-lapse imaging of actin redistribution in an embryo labeled by Tg(actb1::GFP-UtrophinCH) (heatmap). Time in h:min. Scale bar = 50 μm. Related to Fig 7. PSE, pseudostratified epithelium.
(MOV)

## Abbreviations

CMZ: ciliary marginal zone
CNS: central nervous system
ECM: extracellular matrix
FACS: fluorescence-activated cell sorting
hdac1: histone-deacetylase 1
hpf: hours post fertilization
NGS: normal goat serum
n.s.: not significant
PBS-T: PBS with 0.2% or 0.8% Triton X-100
PH3: phosphorylated histone H3
PSE: pseudostratified epithelium
PTU: 1-phenyl-2-thiourea
ROCK: Rho-kinase
ZIRC: Zebrafish International Resource Center
