# Supplementary material for: A non-cell-autonomous actin redistribution enables isotropic retinal growth
Source: PLoS Biol. 2018 Aug 10;16(8):e2006018. doi: 10.1371/journal.pbio.2006018 (PMC6117063; doi:10.1371/journal.pbio.2006018)
Supplement: S1 Table — (PDF) [file pbio.2006018.s001.pdf]

S1 Table: Zebrafish transgenic lines and mutant used in this study.

	Line/ mutant	Labeling/ purpose	Reference
1	Wild-type WT-AB	Control studies	RRID:ZIRC_ZL1
2	Wild-type WT-TL	Control studies	RRID:ZIRC_ZL86
3	Tg(actb1:HRAS-EGFP)	All membranes	[1]
4	Tg(βactin:mKate2-Ras)	All membranes	[2]
5	Tg(actb1:GFP-UtrCH)	F-actin	[3]
6	Tg(hsp70:H2B-RFP)	Chromatin	[4]
7	Tg(ath5:GFP)	Membranes of RGCs and PRs	[2]
8	Tg(ath5:RFP)	Membranes of RGCs and PRs	[2]
9	Tg(SoFa2)	Differentiated retinal cells	[5]
10	hdac1 t24411	Inhibition of cell cycle exit and differentiation in zebrafish retina	[6]

REFERENCES

1. Cooper MS, Szeto DP, Sommers-Herivel G, Topczewski J, Solnica-Krezel L, Kang H-C, et al. Visualizing morphogenesis in transgenic zebrafish embryos using BODIPY TR methyl ester dye as a vital counterstain for GFP. Dev Dyn. Wiley Subscription Services, Inc., A Wiley Company; 2005;232: 359–368. doi:10.1002/dvdy.20252

2. Icha J, Kunath C, Rocha-Martins M, Norden C. Independent modes of ganglion cell translocation ensure correct lamination of the zebrafish retina. The Journal of Cell Biology. Rockefeller University Press; 2016;215: 259–275. doi:10.1083/jcb.201604095

3. Behrndt M, Salbreux G, Campinho P, Hauschild R, Oswald F, Roensch J, et al. Forces driving epithelial spreading in zebrafish gastrulation. Science. 2012;338: 257–260. doi:10.1126/science.1224143

4. Dzafic E, Strzyz PJ, Wilsch-Bräuninger M, Norden C. Centriole Amplification in Zebrafish Affects Proliferation and Survival but Not Differentiation of Neural Progenitor Cells. Cell Rep. 2015;13: 168–182. doi:10.1016/j.celrep.2015.08.062

5. Almeida AD, Boije H, Chow RW, He J, Tham J, Suzuki SC, et al. Spectrum of Fates: a new approach to the study of the developing zebrafish retina. Development. 2014;141: 1971–1980. doi:10.1242/dev.104760

6. Stadler JA, Shkumatava A, Norton WHJ, Rau MJ, Geisler R, Fischer S, et al. Histone deacetylase 1 is required for cell cycle exit and differentiation in the zebrafish retina. Dev Dyn. Wiley-Liss, Inc; 2005;233: 883–889. doi:10.1002/dvdy.20427
