# Supplementary material for: A non-cell-autonomous actin redistribution enables isotropic retinal growth
Source: PLoS Biol. 2018 Aug 10;16(8):e2006018. doi: 10.1371/journal.pbio.2006018 (PMC6117063; doi:10.1371/journal.pbio.2006018)
Supplement: S2 Table — (PDF) [file pbio.2006018.s002.pdf]

S2 Table: Morpholinos used in this study.

	Morpholino (MO)	Injected amount	Sequence 5'-3'	Reference
1	Hdac1	0.5 ng	TTGTTCCTTGAGAACTCAGCGCCAT	[1]
2	P53	1-1.5x of main MO	GCGCCATTGCTTTGCAAGAATTG	[2]

REFERENCES

1. Yamaguchi M, Tonou-Fujimori N, Komori A, Maeda R, Nojima Y, Li H, et al. Histone deacetylase 1 regulates retinal neurogenesis in zebrafish by suppressing Wnt and Notch signaling pathways. Development. The Company of Biologists Ltd; 2005;132: 3027–3043. doi:10.1242/dev.01881

2. Robu ME, Larson JD, Nasevicius A, Beiraghi S, Brenner C, Farber SA, et al. p53 Activation by Knockdown Technologies. PLOS Genet. 2007;3: e78. doi:10.1371/journal.pgen.0030078
