# Supplementary material for: A non-cell-autonomous actin redistribution enables isotropic retinal growth
Source: PLoS Biol. 2018 Aug 10;16(8):e2006018. doi: 10.1371/journal.pbio.2006018 (PMC6117063; doi:10.1371/journal.pbio.2006018)
Supplement: S3 Table — (PDF) [file pbio.2006018.s003.pdf]

S3 Table: Chemical inhibitors/drugs used in this study.

	Chemical	Function	Working dilution	Source/ Cat.No.
1	Trichostatin A (TSA)	Hdac1 inhibition	0.8 mM	Sigma-Aldrich; T1952
2	Rockout	Rho kinase inhibitor	150-175 μM	Santa Cruz; sc-203237
3	Hydroxyurea (HU)	S-phase inhibitor	30 mM	Sigma-Aldrich; H8627-1G
4	Aphidicolin (A)	S-phase inhibitor	150 μM	Sigma-Aldrich; 89458
