# Supplementary material for: A non-cell-autonomous actin redistribution enables isotropic retinal growth
Source: PLoS Biol. 2018 Aug 10;16(8):e2006018. doi: 10.1371/journal.pbio.2006018 (PMC6117063; doi:10.1371/journal.pbio.2006018)
Supplement: S1 Text — (PDF) [file pbio.2006018.s004.pdf]

Supplementary theory notes
A non-cell autonomous actin redistribution governs tissue shape maintenance during retinal growth

Marija Matejcic, Guillaume Salbreux, and Caren Norden

In this supporting notes we discuss a set of equations that describe the key aspects of the retina growth between 20 and 48hpf. We take into account balance equations that correspond to the change in number of undifferentiated and neuron or committed precursor cells, and to how the volume growth of the retina is decomposed in cell volume change and change in the number of cells. We then introduce a simple description of force balance in the tissue taking into account surface tensions generated along the cellular lateral interfaces, and line tensions generated apically and basally. Taking into account the cell division and differentiation rate, the rate of volume growth, and the change in ratio of lateral surface tension to apicobasal line tension, allows to close the system of equations to obtain a description of the change in time of retina volume, height, area and number of cells.

I. BALANCE EQUATIONS

We consider two cell types, undifferentiated progenitors (P) and neurons or committed precursors (N). We assume that progenitors divide with a rate k. Each division yields two undifferentiated progenitors with probability p, two neurons or committed precursor with probability $(1-p)\alpha$ and one progenitor and one neuron/committed precursor with probability $(1-p)(1-\alpha)$ (S4 Fig. A). Denoting N_p and N_n the number of progenitors and neurons/committed precursors in the retina, and $N_t = N_p + N_n$ the total number of cells, we write the following kinetic equations for the number of cells:

$$\frac{dN_p}{dt} = kpN_p - k(1-p)\alpha N_p = k(p + \alpha p - \alpha)N_p \tag{1}$$

$$\frac{dN_n}{dt} = k(1-p)(1-\alpha)N_p + 2k(1-p)\alpha N_p = k(1-p)(1+\alpha)N_p \tag{2}$$

$$\frac{dN_t}{dt} = kN_p \quad . \tag{3}$$

The cell doubling time T_{cc} is related to the rate of cell division k by $T_{\mathrm{cc}} \simeq \ln 2/k$. We find that the mean cell cycle length is roughly constant over time, with an average cell doubling time $T_{\mathrm{cc}} \simeq 6.2$h (S4 Fig. C). This corresponds to an average rate of cell division

$$k \simeq 0.11h^{-1}. \tag{4}$$

We further make the simplifying approximation here that the number of asymmetric divisions can be neglected, $\alpha = 1$. We also assume that no neuron or committed precursor are present before 35h, as only a low number of differentiated cells can be detected in the retina during this time. Therefore, we assume $p = 1$ prior to 35hpf. Adjusting for experimental data of the number of differentiated neurons and total number of cells after this time point, we find $p \simeq 0.65$. Specifically, we fitted experimental data of $N_t(t)$ and $N_n(t)$ with $N_t(20\text{hpf})$ and $p(t > 35\text{hpf})$ as free parameters. The function $p(t)$ is then given by:

$$p(t) = \begin{cases} 1 & t < 35\text{hpf} \\ 0.65 & t > 35\text{hpf} \end{cases} \quad . \tag{5}$$

Note that here we chose to do a simple fit to the data taking into account a step function of probability p, but the actual value of $p(t)$ might change in time with a more complicated dynamics [1].

The average cell volume v increases due to the overall increase of volume of the tissue, and decreases because of cell division. This can be expressed through the balance equation

$$\frac{1}{v}\frac{dv}{dt} = \frac{1}{V}\frac{dV}{dt} - \frac{1}{N_t}\frac{dN_t}{dt} \tag{6}$$

where V is the total volume of the retina. We find that the change of total volume is well described by an exponential growth with rate

$$g = \frac{1}{V}\frac{dV}{dt} \simeq 0.055\text{h}^{-1}, \tag{7}$$

where the rate in Eq. 7 is obtained from a fit of experimental data to the function $\ln V(t) = gt + V_0$, with g and V_0 as free parameters (S4 Fig. C).

Eq. 6 can be integrated to yield

$$\ln \frac{V(t)}{V(t_0)} = \ln \frac{N_t(t)}{N_t(t_0)} + \ln \frac{v(t)}{v(t_0)} \quad , \tag{8}$$

with $t_0 = 20$hpf. Different terms in Eq. 8 are plotted in Fig. 2H and S4 Fig. D from experimental data to obtain a decomposition of tissue volume growth into average cell volume growth and cell number change. Similarly, in Fig. 2G and S4 Fig. D we plot different terms of the decomposition:

$$\ln \frac{V(t)}{V(t_0)} = \ln \frac{S(t)}{S(t_0)} + \ln \frac{h(t)}{h(t_0)} \quad , \tag{9}$$

with $S(t)$ the tissue average surface area and $h(t)$ the tissue height. Here we make the simplifying assumption that the tissue volume is linearly dependent in the tissue average area and the tissue height.

II. CELL SHAPE AND MECHANICAL EQUATIONS

We consider here a simple model of cell geometry where cells are described by truncated cones with apical and basal surface area s_a and s_b, height h, and radius in the middle plane r (S4 Fig. B). The middle plane is defined here as the plane with cross-sectional surface area equal to the mean apical and basal surface areas. We do not discuss the origin of cell and tissue curvature, which is captured in a relative area parameter $s = s_a/s_b$, taken to be constant and equal on average to ~ 4.4 from experimental data (S4 Fig. C). The cell apical area is given by $s_a = \pi r_a^2 = \frac{2s}{1+s}\pi r^2$ and the basal area is given by $s_b = \pi r_b^2 = \frac{2}{1+s}\pi r^2$. As a result, the radii of the apical and basal cell surfaces are given by $r_a = \sqrt{\frac{2s}{1+s}}r$ and $r_b = \sqrt{\frac{2}{1+s}}r$, the apical and basal perimeters by $p_a = 2\pi r_a$ and $p_b = 2\pi r_b$, the lateral surface area by $s_l = \pi(r_a + r_b)\sqrt{(r_a - r_b)^2 + h^2}$. The cell volume v is given by

$$v = \frac{\pi}{3}(r_a^2 + r_b^2 + r_a r_b)h = \frac{2\pi(1 + s + \sqrt{s})}{3(1+s)}r^2 h \quad . \tag{10}$$

To obtain the total average tissue area, we consider for simplicity that the retina is made of identical cells, such that:

$$S = N_t \pi r^2 = \frac{3(1+s)N_t v}{2(1+\sqrt{s}+s)h} \quad . \tag{11}$$

Deviations from this ideal behaviour, for instance due to the fact that cells of the retina margin have different shapes than cells in the central part of the retina, may be at the origin of differences between the area predicted by the simplified model and experimentally measured areas (Fig. 5G and S4 Fig. D).

To describe cell mechanics, we consider a simple model where cells have line tensions around the apical and basal perimeter Λ_a and Λ_b, and surface tension along their lateral surface T_l. The line tensions Λ_a can arise from the apical actomyosin epithelial belt, the line tensions Λ_b from the basal actomyosin belt. The surface tension T_l can arise from the actomyosin cortex exerting tension along cellular interfaces. The mechanical energy for one cell is then written

$$W = \Lambda_a p_a + \Lambda_b p_b + T_l s_l - P\left(\frac{2\pi(1+s+\sqrt{s})}{3(1+s)}r^2 h - v\right) \quad , \tag{12}$$

where geometrical quantities have been introduced above, and P is a Lagrange multiplier enforcing the volume constraint and corresponding to the intracellular pressure. Minimizing W with respect to r, h and P, one obtains the equilibrium cell height written here in the limit of highly columnar cells, $h \gg r$:

$$h_{\text{eq}} \simeq 2\frac{\sqrt{s}\Lambda_a + \Lambda_b}{(\sqrt{s}+1)T_l} \tag{13}$$

We further assume that dissipative contributions slow down the height relaxation on a time scale τ_h, such that

$$\frac{dh}{dt} = -\frac{1}{\tau_h}(h - h_{\text{eq}}) \quad . \tag{14}$$

Therefore, in this simple model, the cell height is set by the ratio $\frac{(\sqrt{s}\Lambda_a+\Lambda_b)}{T_l}$. Actin intensity measurements (Fig. 5B) indicate that the ratio of apical and basal actin to lateral actin concentration increases after 36hpf. We assume here that observed changes in these actin ratio lead to a change in the ratio of apicobasal line tensions and lateral surface tensions, such that:

$$\frac{(\sqrt{s}\Lambda_a + \Lambda_b)}{T_l}(t) = \begin{cases} 67\mu\text{m} & t < 36\text{hpf} \\ 101\mu\text{m} & t > 36\text{hpf} \end{cases} . \tag{15}$$

where these values were chosen to match the experimentally measured cell height change (Fig. 5E). Note that actomyosin distribution has a varying spatial profiles along lateral interfaces (Figs. 4C-E), that we do not discuss here for simplicity.

III. TISSUE GROWTH DYNAMICS

Combining the equations 1-7 and the force balance equations 13-14 with the apico-basal line tension to lateral surface tension ratio given by equation 15, we can solve for the evolution in time of the parameters characterizing tissue growth. We plot in Fig. 3F, Fig. 5E-5G and S4 Fig. D the resulting curves for the total number of cells and the number of neurons, the cell and tissue volume, the tissue height and the tissue area. Parameters are determined from fits to experimental data, except for τ_h which we take equal to 3h, as the data time resolution does not allow for a more precise determination. Initial conditions at $t = 20$hpf are determined from experimental data or fits to experimental data. The agreement between the simplified theory and experimental data corresponds to the following picture of retinal growth:

- Tissue volume growth and progenitor cell division occur at a roughly constant rate.
- Before 35hpf, there is no cell differentiation, and the actomyosin distribution is maintained, such that forces generated by the actomyosin cytoskeleton ensure a constant tissue height. Because the rate of volume growth $g \simeq 0.055\text{h}^{-1}$, is about twice lower than the rate of cell division $k \simeq 0.11h^{-1}$, the cell volume is decreasing (Fig. 5F).
- After 35hpf, $\sim$ 35% of cell divisions lead to cell differentiation. At about the same time, the actomyosin distribution is changing, leading to cell elongation. The relative rate of new cell production slows down, due to terminally differentiated cells stopping division. As a result, the relative rate of cell volume decrease is also reduced.

In Fig. 6G we plot the tissue aspect ratio calculated from the equations described in section II, as well as for a modified model where the ratio $\frac{(\sqrt{s}\Lambda_a+\Lambda_b)}{T_l}$ does not change and is equal to 67μm. This corresponds to a situation where the actomyosin distribution does not change, and as a result the cell height is constant. In that case the tissue aspect ratio keeps decreasing after 36hpf (Fig. 6G).

---

[1] Jie He, Gen Zhang, Alexandra D Almeida, Michel Cayouette, Benjamin D Simons, and William A Harris. How variable clones build an invariant retina. *Neuron*, 75(5):786–798, 2012.
