# Supplementary figures and images for: A non-cell-autonomous actin redistribution enables isotropic retinal growth
Source: PLoS Biol. 2018 Aug 10;16(8):e2006018. doi: 10.1371/journal.pbio.2006018 (PMC6117063; doi:10.1371/journal.pbio.2006018)

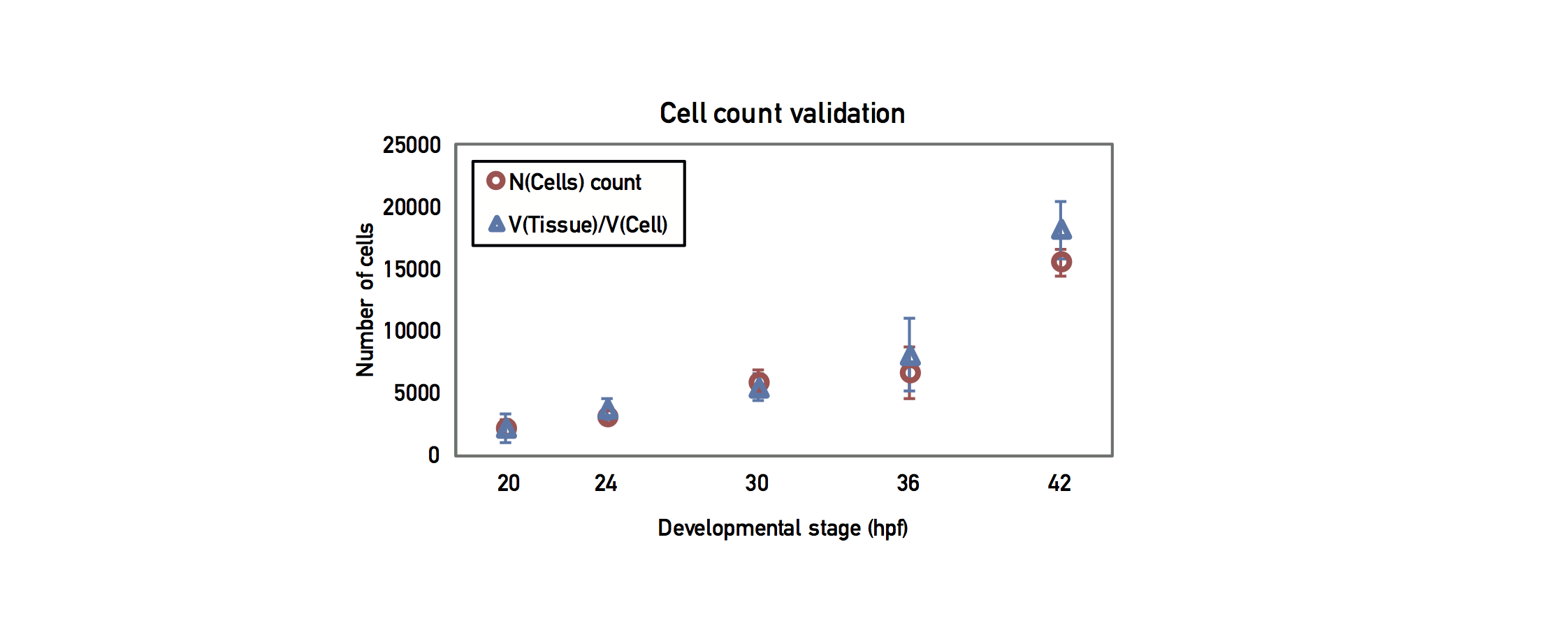

Supplement: S1 Fig — (A) The cell count (red) was validated using mitotic cell volumes by dividing the average tissue volume by 90% of mitotic cell average volume for each stage (see Materials and methods). Data are plotted as stage mean ± SD. n = 10 samples/stage for all. (Underlying data can be found at DOI: 10.5281/zenodo.1316912; /Matejcic-et-al_2018/Data/F1_2_3D_S12BD34.csv.). (TIFF) [file pbio.2006018.s005.tiff]

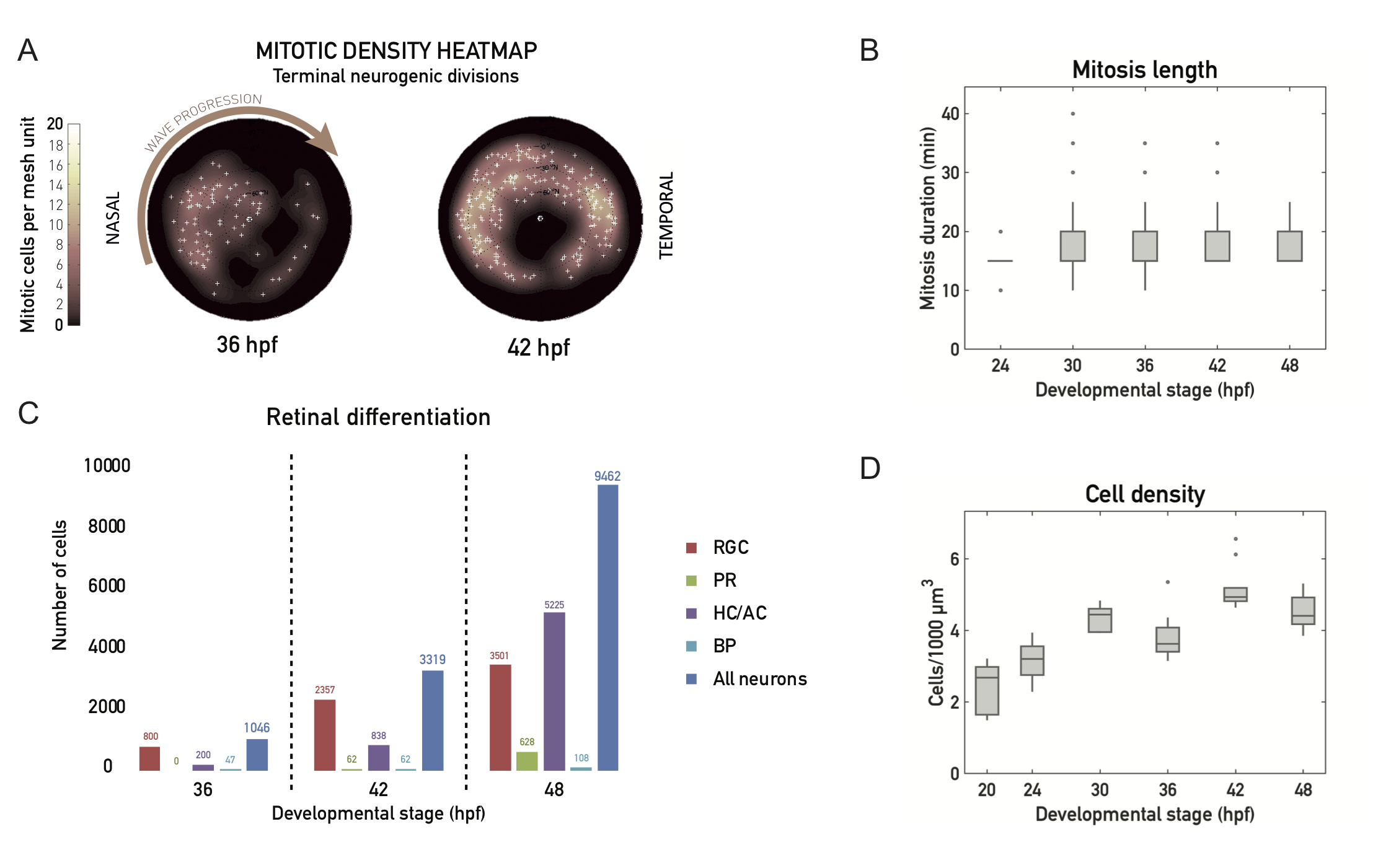

Supplement: S2 Fig — (A) Heatmaps of Ath5+ (neurogenic) mitotic divisions at 36 hpf and 42 hpf. This validation shows that nonuniform cell divisions are detectable using the method in Fig 3A. Neurogenic divisions are spatially nonuniform, progressing through the tissue as a naso-temporal wave. (B) Duration of mitosis does not change over development. Cells labeled mosaically with Hsp70::H2B-RFP or Hsp70::EGFP-PCNA were tracked in light sheet time lapses at 5 min time resolution. Data were binned as developmental stage +/− 3 h. N = 197 cells from 20 embryos (24 hpf N = 20; 30 hpf N = 56; 36 hpf N = 57; 42 hpf N = 53; 48 hpf N = 11). (C) Retinal neurogenesis. Average number of neuronal subtypes, as analyzed by FACS from pooled dissected Tg(SoFa) retinal samples. N = 20 retinas/stage. Data were normalized to wild-type background fluorescence. (D) Cell density was calculated by dividing the number of cells by total tissue volume. N = 10 samples/stage. (Underlying data can be found at DOI: 10.5281/zenodo.1316912; for panels B and D at /Matejcic-et-al_2018/Data/F1_2_3D_S12BD34.csv, panel C at S2C.xlsx.). Ath5, atonal homolog 5; FACS, fluorescence-activated cell sorting; hpf, hours post fertilization. (TIFF) [file pbio.2006018.s006.tiff]

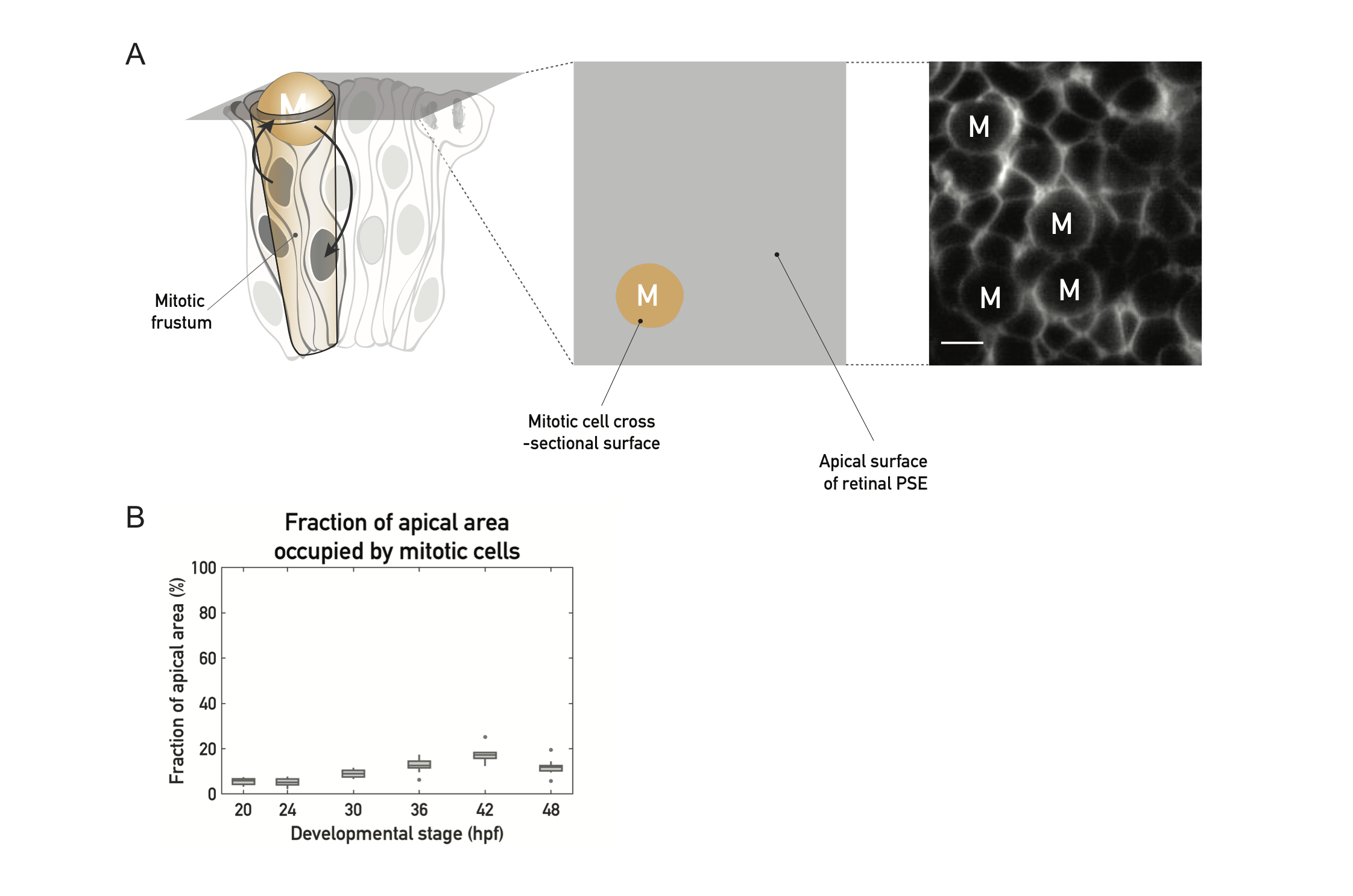

Supplement: S3 Fig — (A) Left: Schematic representation of PSE tissue architecture, with apical mitoses, migrating nuclei (arrows), and the mitotic frustum. The mitotic frustum is depicted as a conical unit below the rounded mitotic cell. We assume that all interphase nuclei in a single mitotic frustum (gray ellipses) undergo mitosis at the same position at the apical surface (gray). Middle: Schematic top view onto the apical surface cross-section (gray plane) marked in the left schematic. Interphase cells’ apical attachments are not shown. Right: Apical surface of the retinal PSE at 35 hpf, with cross-sections of mitotic and interphase cells. Cell membranes are labeled with Tg(actb1:HRAS-EGFP). Frame from Video 2. M: mitotic cells. Scale bar: 10 μm. (B) Fraction of the apical tissue surface area occupied by mitotic cells; 10 samples/stage. Related to Fig 3G. (Underlying data can be found at DOI: 10.5281/zenodo.1316912; /Matejcic-et-al_2018/Data/F1_2_3D_S12BD34.csv.). hpf, hours post fertilization; PSE, pseudostratified epithelium. (TIFF) [file pbio.2006018.s007.tiff]

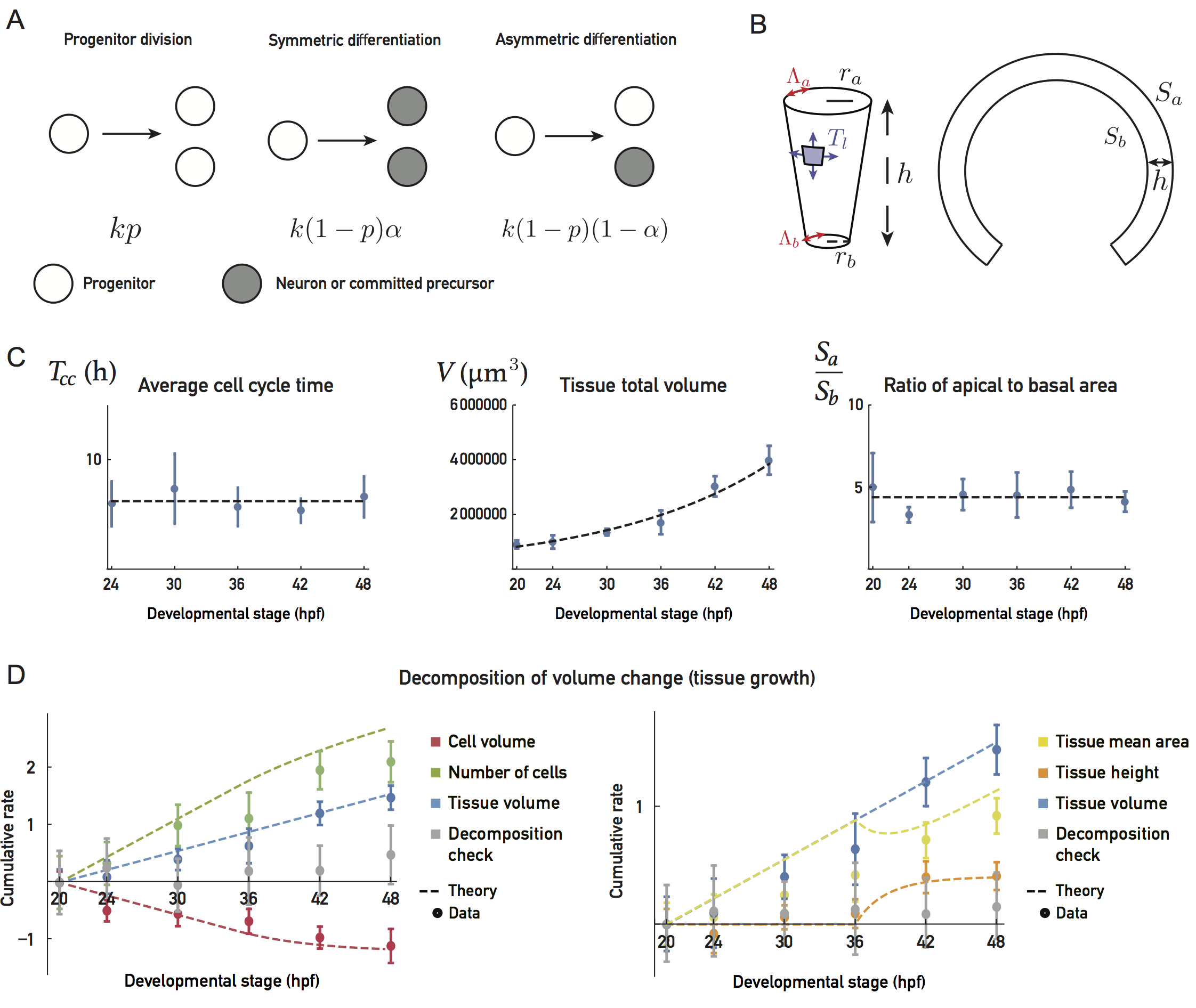

Supplement: S4 Fig — (A) Schematic of division and differentiation rules considered in the simplified description of retina growth. For simplicity, we consider 2 cell populations—progenitors (white) and neurons, or committed precursors (gray). Progenitors divide with a constant rate k. Cell division give rise to 2 progenitors with probability p, 2 neurons/committed precursors with probability (1−p)α, 1 progenitor, and 1 neuron/committed precursor with probability (1−p)(1−α). Here, we assume that only symmetric differentiation events occur, such that α = 1. (B) Schematic of cell and tissue shape geometry. Cells are represented by truncated cones with apical and basal line tensions Λa and Λb, and lateral surface tension Tl. The apical and basal tissue surface area is obtained in the simplified description by multiplying the cellular apical and basal area by the number of cells. (C) Experimental data for the cell cycle time, tissue volume and ratio of apical to basal surface area as a function of time, and fit to a constant average value (cell cycle time and ratio of areas) or to an exponential (tissue volume). (D) Comparison between experimentally measured cumulative logarithmic rates of change of tissue volume, number of cells, cell volume, tissue area and tissue height, and cumulative logarithmic rates obtained from the simplified description. (Underlying data can be found at DOI: 10.5281/zenodo.1316912; /Matejcic-et-al_2018/Data/F1_2_3D_S12BD34.csv. The theoretical analysis can be found at /Matejcic-et-al_2018/Theory/Data analysis_essentials.nb.). hpf, hours post fertilization. (TIFF) [file pbio.2006018.s008.tiff]

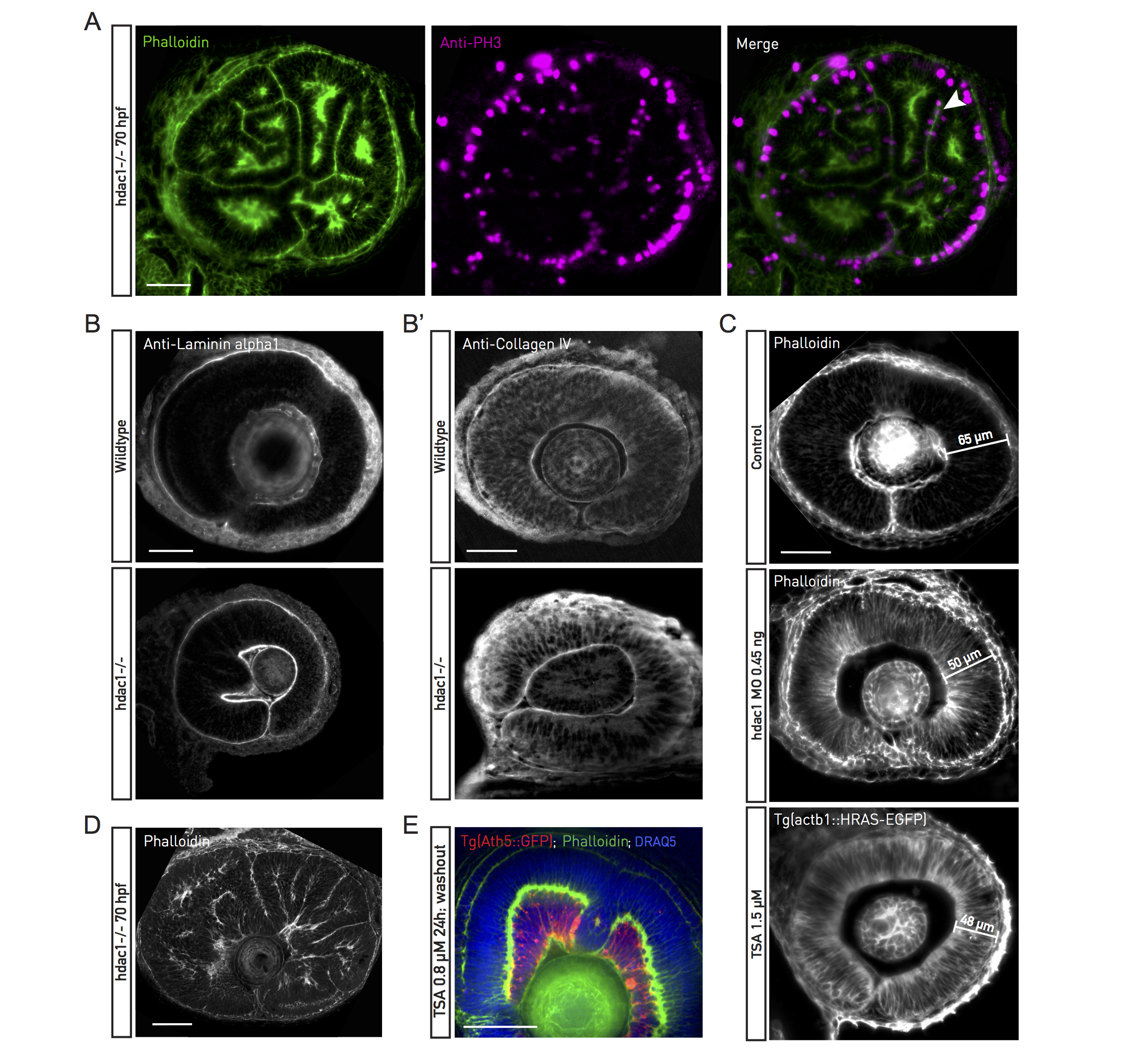

Supplement: S5 Fig — (A) Phalloidin (green) and PH3 antibody (magenta) staining of F-actin and mitotic cells in about 70 hpf hdac1−/− retinal PSE. Apical mitoses, junctional belts, and basal actin accumulation are preserved in the folded tissue. (B) Laminin-alpha1 and Collagen IV (B’) antibody staining of wild-type and hdac1−/− retinal tissues around 48 hpf. An intact basal lamina underlies the basal surface of the hdac1−/− retinal PSE. (C) Tissue thickness does not increase in Hdac1 morpholino-injected or TSA-treated retinal PSE (42 hpf). (D) Phalloidin staining of approximately 72 hpf hdac1−/−. Epithelial folds form throughout the retinal PSE. (E) Tg(Ath5::GFP) retinas (red) treated with TSA at 24 hpf for 24 h and stained with phalloidin (green) and DRAQ5 (blue). The medium was replaced after 24 h. The PSE differentiates by 70 hpf despite tissue shape perturbed by folds. Consequently, neuronal layers are perturbed, as well. Scale bars: 50 μm for all images. DRAQ5, deep red anthraquinone; Hdac1, histone-deacetylase 1; hpf, hours post fertilization; PH3, phosphorylated histone H3; PSE, pseudostratified epithelium; TSA, Trichostatin-A. (TIFF) [file pbio.2006018.s009.tiff]

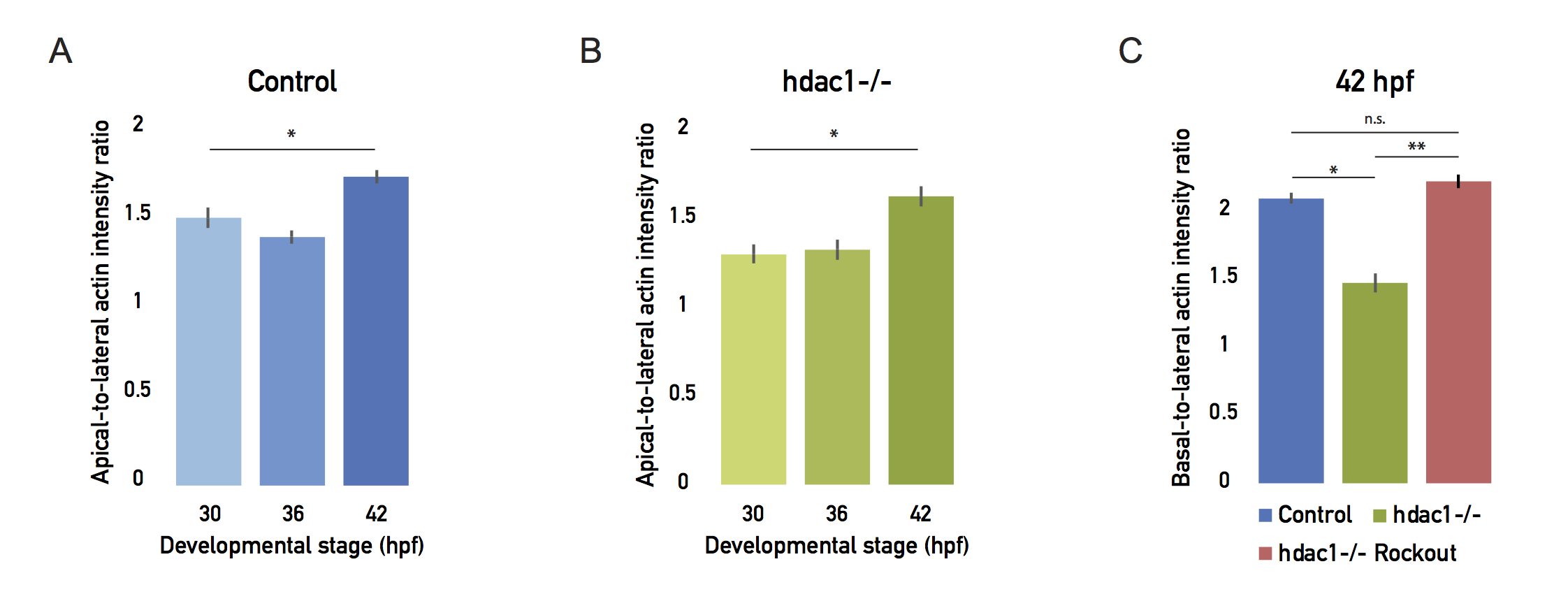

Supplement: S6 Fig — (A) Ratios of apical-to-lateral phalloidin signal intensity in control hdac1+/− tissue over development; 5 samples/stage. Mean ± SD. Mann-Whitney test, p-value 0.0317. (B) Ratios of apical-to-lateral phalloidin signal intensity in hdac1−/− tissues over development; 5 samples/ stage. Mean ± SD. Mann-Whitney test, p-value 0.0159. (C) Ratios of basal-to-lateral phalloidin signal intensity at 42 hpf in control and hdac1−/− tissues (N = 5) and in hdac1−/− tissue treated with 150 μM Rockout (N = 6). Rockout treatment abolishes the basolateral actin accumulation in hdac1−/− and restores the basal-to-lateral actin ratio to control values. Mean ± SD. Mann-Whitney test, control versus hdac1−/− p-value 0.0317; hdac1−/− versus hdac1−/− Rockout p-value 0.0043; control versus hdac1−/− Rockout p-value 0.4127. (Underlying data can be found at DOI: 10.5281/zenodo.1316912; /Matejcic-et-al_2018/Data/F5B_6D_S6.csv.). hdac1, histone-deacetylase 1; hpf, hours post fertilization. (TIFF) [file pbio.2006018.s010.tiff]

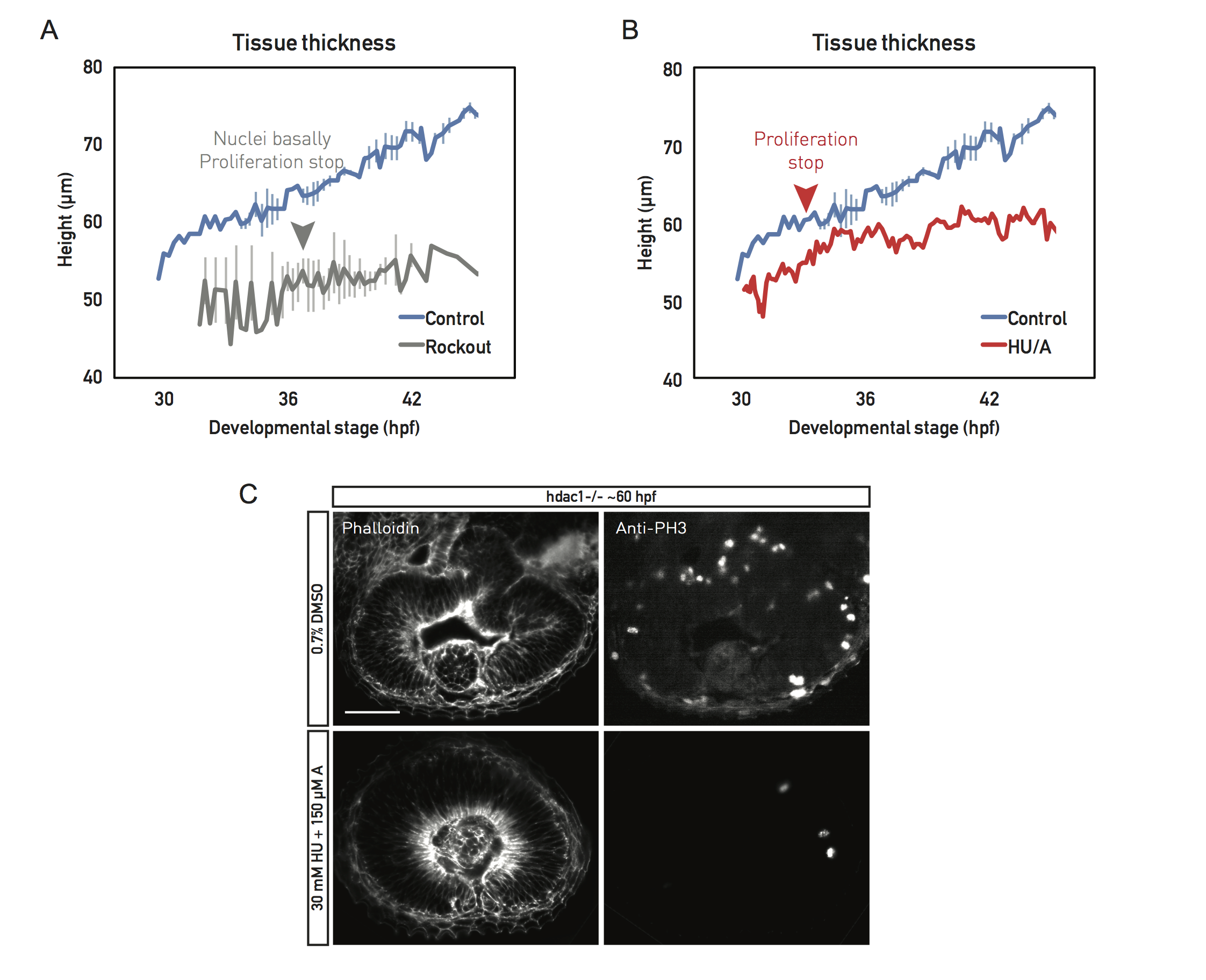

Supplement: S7 Fig — (A) Cell height of live (light sheet time lapses) Rockout-treated wild-type retinal PSE (gray). Sample was incubated in 175 μM Rockout for the entirety of the imaging session. Around 37 hpf, the basolateral actin accumulation was abolished, nuclei filled the basal positions, and proliferation stopped. Related to S6 Movie. (B) Cell height of live (light sheet time lapses) wild-type retinal PSE, treated with a combination of cell cycle inhibitors HU/A (red); 30 mM HU and 150 μM of A were added at the beginning of the movie (30 hpf). Proliferation stopped 3 h later (red arrowhead). Cell height did not increase further after cells cycle was blocked. Control plots (blue) in (A) and (B) are data from Fig 6E. (C) Top panels: Retinas of mutant fish treated with DMSO fold and proliferate normally (PH3 staining). Bottom panels: Mutant retinas treated with HU/A do not fold when their proliferation is inhibited but do maintain the basolateral actin accumulation. Scale bar: 50 μm. (Underlying data can be found at DOI: 10.5281/zenodo.1316912; /Matejcic-et-al_2018/Data/FS7A.csv and FS7B.csv.). A, aphidicolin; hpf, hours post fertilization; HU, hydroxyurea; PH3, phosphorylated histone H3; PSE, pseudostratified epithelium. (TIFF) [file pbio.2006018.s011.tiff]
